# ProteinCoLoc streamlines Bayesian analysis of colocalization in microscopic images

**DOI:** 10.1038/s41598-024-63884-1

**Published:** 2024-06-10

**Authors:** Manuel Seefelder, Stefan Kochanek, Fabrice A. C. Klein

**Affiliations:** Department of Gene Therapy, Ulm University, 89081 Ulm, Germany

**Keywords:** Huntington's disease, Fluorescence imaging, Computational biology and bioinformatics, Software

## Abstract

Colocalization, the spatial overlap of molecular entities, is often key to support their involvement in common functions. Existing colocalization tools, however, face limitations, particularly because of their basic statistical analysis and their low-throughput manual entry processes making them unsuitable for automation and potentially introducing bias. These shortcomings underscore the need for user-friendly tools streamlining colocalization assessments and enabling their robust and automated quantitative analyses. We have developed ProteinCoLoc, an innovative software designed for automated high-throughput colocalization analyses and incorporating advanced statistical features such as Bayesian modelling, automatic background detection and localised correlation analysis. ProteinCoLoc rationalises colocalization assessments without manual input, comes with a user-friendly graphical user interface and provides various analytics allowing to study and locally quantify colocalization. This easy-to-use application presents numerous advantages, including a direct comparison with controls employing a Bayesian model and the analysis of local correlation patterns, while reducing hands-on time through automatic background detection. The software was validated while studying the colocalization pattern of two proteins forming a stable complex: the huntingtin protein (HTT) and its partner huntingtin-associated protein 40 (HAP40). Our results showcase the software’s capacity to quantitatively assess colocalizations. ProteinCoLoc is available both as a Julia package and as a compiled software (https://github.com/ma-seefelder/ProteinCoLoc).

## Introduction

The term colocalization refers to the spatial overlap of two or more entities, such as proteins, molecular probes, or cellular structures, within a given biological sample. Studying the colocalization is of major importance to improve our understanding of cellular processes. Therefore, immunocytochemistry (ICC)^1^ has emerged as a powerful and widely applied method in cellular and molecular biology that allows studying the colocalization between two or more fluorescently labelled probes, e.g. proteins.

Contemporary colocalization analysis tools and software packages developed to quantitatively study the colocalization between two proteins rely on correlation analysis between pixel intensities^2,3^. Despite being widely used and fairly effective, those tools have important limitations, such as requiring users to capture images with minimal background and manually annotate the background^4^. This labour-intensive process is a bottleneck in high-throughput settings and the analysis may introduce bias due to the potential subjectivity of manual input. Furthermore, existing tools primarily calculate colocalization for entire images, lacking the direct capability to test for local colocalization, whereas functionally relevant colocalization events may occur only in defined cellular regions. Another pitfall lies in the statistical analysis of colocalization, which is not a trivial task as the intensity of a pixel often depends on the intensity of the surrounding pixels (autocorrelation). For this reason, existing methods that rely on a Student’s t-test may exaggerate the reliability of colocalizations, as such a test yields a deflated, i.e. systematically lower, *p* value given the auto-correlation between promiscuous pixels^3^. As an alternative approach, pixels or blocks of pixels are often scrambled either individually or in groups to retain autocorrelation and more accurately estimate the true probability distribution of the null hypothesis, which is defined as the absence of colocalization^3^.

Having highlighted the limitations of contemporary colocalization analysis tools, it becomes evident that there is a critical need within the scientific community for an advanced and user-friendly tool capable of not only streamlining colocalization assessments but also facilitating robust quantitative analyses. In response to this need, we developed ProteinCoLoc, an innovative software application designed to overcome the limitations of existing tools and provide a comprehensive solution for quantitative protein colocalization analysis. For example, ProteinCoLoc automatically detects background pixels by Otsu’s thresholding^5^ reducing the need for extensive user input and hands-on time required for analysis, making ProteinCoLoc well-suited for studies involving large datasets or automated experimental setups. Furthermore, we implemented tools to detect local correlation patterns and to perform statistical inferences by incorporating a hierarchical Bayesian model instead of the Student’s T-test. Importantly, ProteinCoLoc is also easy to use as it does not require programming skills and comes with a user-friendly graphical user interface (GUI), which includes the functionality to compare the results with control images as well as a plethora of automatically generated plots that summarise and facilitate the interpretation of the results.

To illustrate the applicability and advantages of ProteinCoLoc, we performed two exemplary analyses. In the first analysis, we did a co-immunocytochemistry staining of the huntingtin-associated protein 40 (HAP40) with two different commercial antibodies detecting different epitopes. As both antibodies detect the same protein, a high colocalization, especially in the cell nucleus^6,7^, is expected. In the second analysis, we co-stained HAP40 and huntingtin (HTT), two proteins, whose interaction is well documented^8^ and which are routinely studied in our lab^8,10,12,15,16^. HAP40 is an abundant interactor of HTT^10,11^, the protein that is pathologically altered in Huntington disease (HD) due to a mutation in the *HTT* gene. The high stability of the HAP40-HTT interaction enabled us to resolve the structure of the complex^10,12–14^. Moreover, the interaction of the two proteins is likely essential for their function, as both proteins have co-evolved and their interaction is conserved in various species^15^. Interestingly, the protein levels and stability of both HAP40 and HTT are reduced in tissues from HD patients and mouse models^9,16–18^, suggesting that the reduction of their levels in cells may contribute to the pathophysiology of HD^8,18^. However, although the HTT-HAP40 complex is well characterised in vitro^10,12,15,18^, little is known regarding the extent of their interaction in vivo. Since HTT interacts with hundreds of partners^19,20^, both proteins may also have independent functions and only partially co-localise in cells. For all these reasons, HTT and HAP40 constituted technically ideal and scientifically relevant partners serving as a model to quantify their colocalization using ProteinCoLoc.

We here show that ProteinCoLoc represents a comprehensive solution for protein colocalization analysis, addressing key limitations in current tools through (1) integration of a hierarchical Bayesian model to compare the colocalisation between two biological groups, (2) automatic background detection, (3) implementation of tools to investigate local colocalisation patterns and (4) a user-friendly graphical user-interface (GUI). This fusion of advanced statistical capabilities and accessible interface permits straightforward analysis of colocalization patterns without the need for programming expertise. Furthermore, the accessibility of the source code under a permissible open-source license allows adaptation for high-throughput automated studies, which will make ProteinCoLoc a valuable tool for researchers seeking precision and efficiency in the study of cellular processes.

## Results

In the following two sections, we delineate the outcomes of two exemplary analyses conducted with ProteinCoLoc, illustrating the insights that can be derived from its use. The supplementary materials include all original microscopy images (Supplementary files S1 and S2), a Jupyter notebook (Supplementary file S3), and a step-by-step manual (Supplementary file S4), enabling readers to comprehensively replicate the analyses. The implementation and methodology of ProteinCoLoc are comprehensively detailed in the Methods section.

### Exemplary analysis 1: colocalization between two HAP40 polyclonal antibodies

For the first exemplary analysis, A549 cells were transfected with a plasmid for full-length human HAP40 with a carboxy-terminal TwinStrep-tag, controlled by a human cytomegalovirus (hCMV) promoter^10^. A negative control involved A549 cells with an empty plasmid. Detection employed polyclonal anti-HAP40 (Santa Cruz, sc-69489) and monoclonal anti-Strep (IBA, 2–1507-001) antibodies. For the statistical analysis, six images of the cells expressing recombinant HAP40 and of the negative control were captured by confocal laser-scanning microscopy (Leica TCS SP8) (Supplementary file S1). Bayesian analysis was conducted with 10,000 iterations, 100,000 posterior samples, 16 × 16 patches, and a Δρ threshold of 0.1.

**Figure 1:**
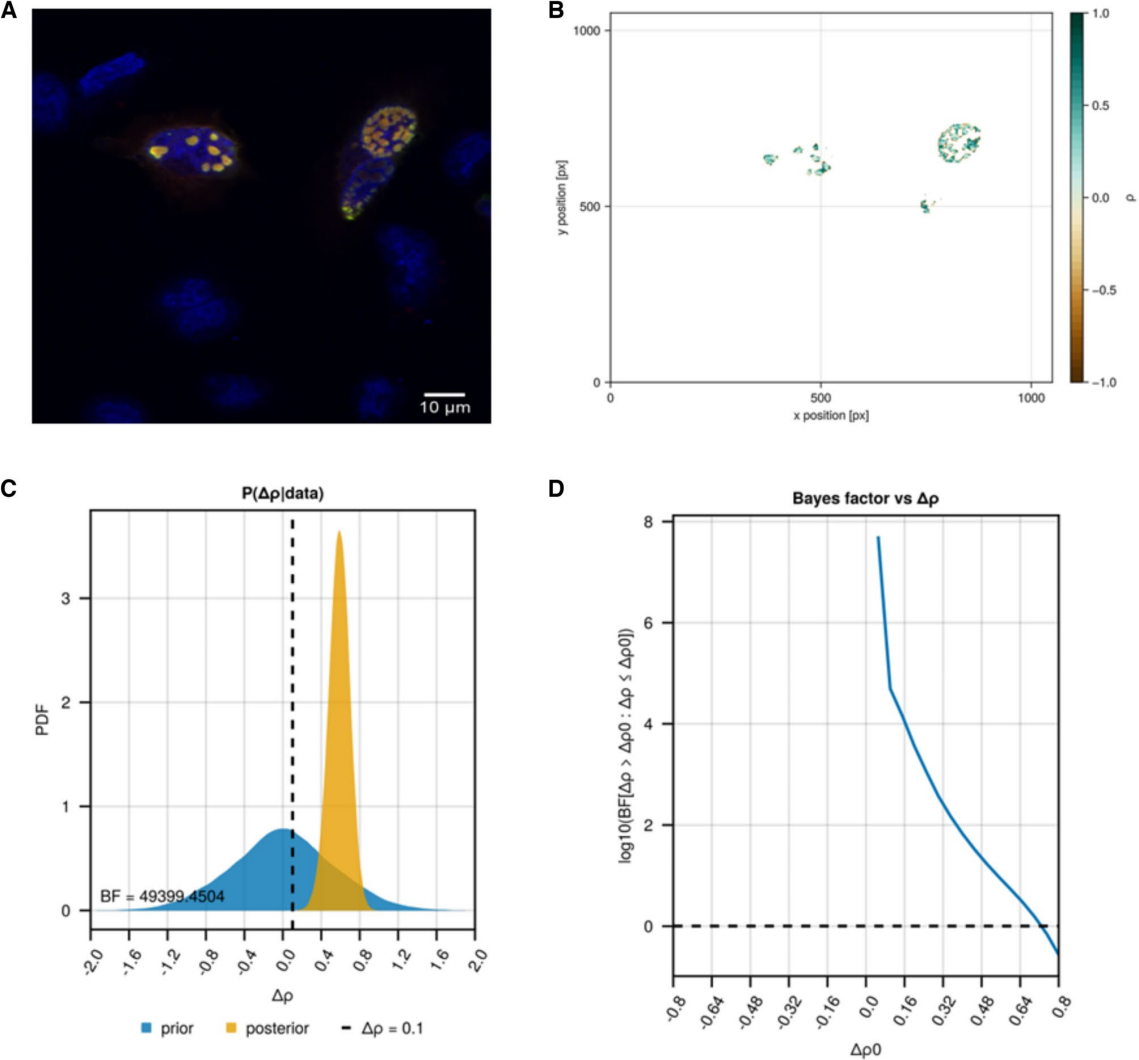
Result of exemplary analysis 1—colocalization between two polyclonal antibodies binding HAP40. (**A**) Co-staining of HAP40 with the anti-HAP40 antibody sc-69489 (Donkey anti-goat Alexa Fluor 555, red) and anti-Strep antibody (Donkey anti-mouse Alexa-Fluor 468). DNA was stained with DAPI and is depicted in blue. (**B**) Local correlation plot between the signal from the anti-HAP40 antibody sc-69489 and anti-Strep antibody. (**C**) Bayes factor plot displaying the prior and posterior distribution for the global ${\Delta }\rho$ between control images (i.e. non-transfected cells, N = 6) and the images from the co-staining (N = 6). (**D**) Bayes factor range plot displaying the Bayes factor at different thresholds for ${\Delta }\rho$.

**Figure 2:**
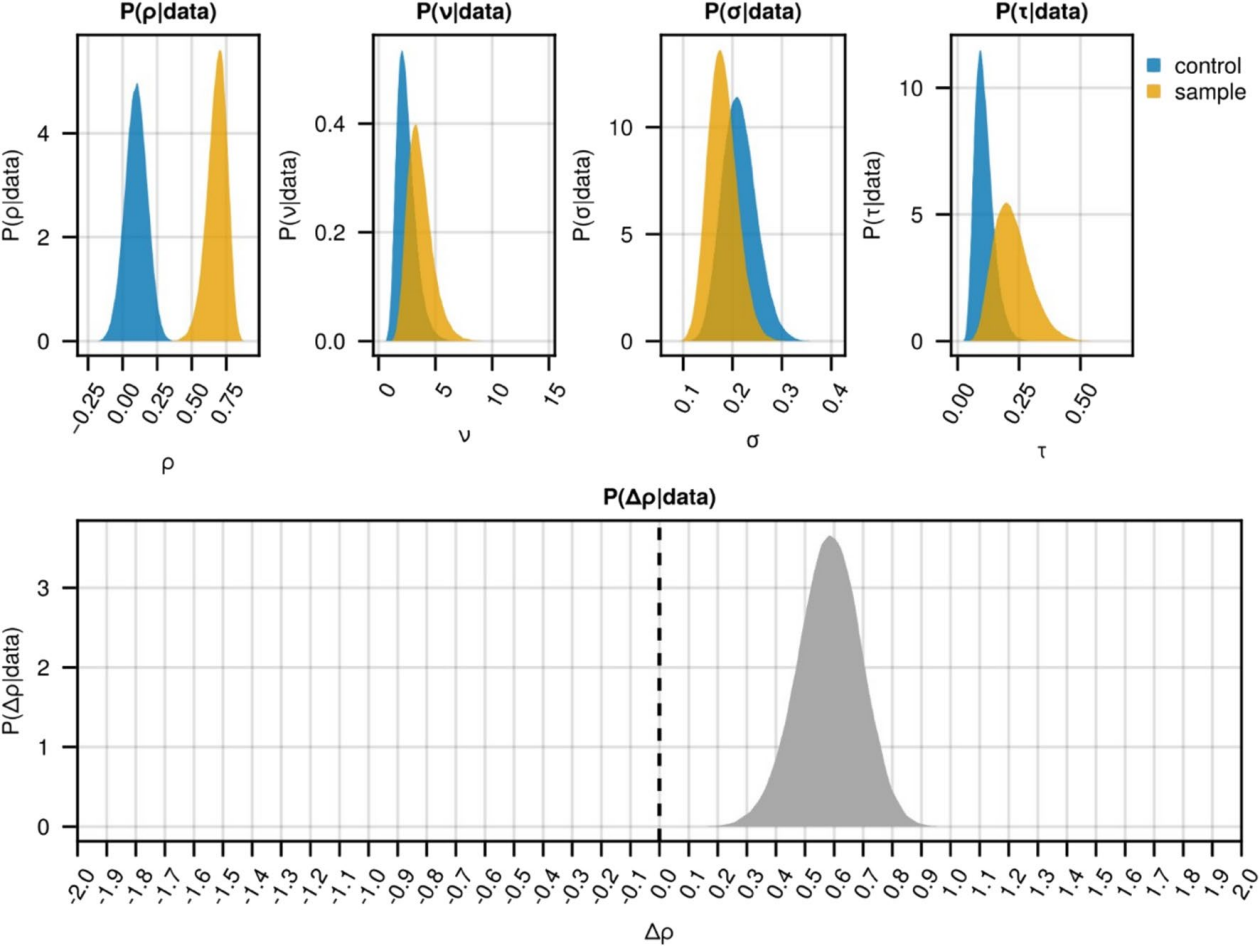
Plot of the posterior distributions of all global model parameters for the control images and the images where HAP40 was co-stained with two distinct antibodies (Fig. 1). ${\uprho }$: global Pearsons correlation coefficient; $\nu$: degrees of freedom; $\sigma$: variability of ${\uprho }$ within images; $\tau :$ standard deviation of ${\uprho }$ between images.

The merged image (Fig. 1A) shows a significant overlap of signals within the nucleoplasm for HAP40 transfected cells, unlike the control (Supplementary file S1). A local correlation analysis (Fig. 1B) confirmed a strong positive correlation in nuclear focal structures, where overexpressed HAP40 was already reported to localise^6,7^. Statistical analysis using the implemented Bayesian hierarchical model provides extreme evidence for significant colocalization (Δρ > 0.1) of the signals with a Bayes factor of 49,399.45:1 (Fig. 1C). The relationship between the Bayes factor and Δρ, along with prior and posterior distributions of Pearson’s correlation coefficients ρ, are detailed in Figs. 1D and 2, respectively. Posterior mean of ρ for HAP40 and control cells were estimated at 0.680 ([0.519; 0.804] 95% credible interval) and 0.096 ([− 0.063; 0.245] 95% credible interval), respectively.

**Figure 3:**
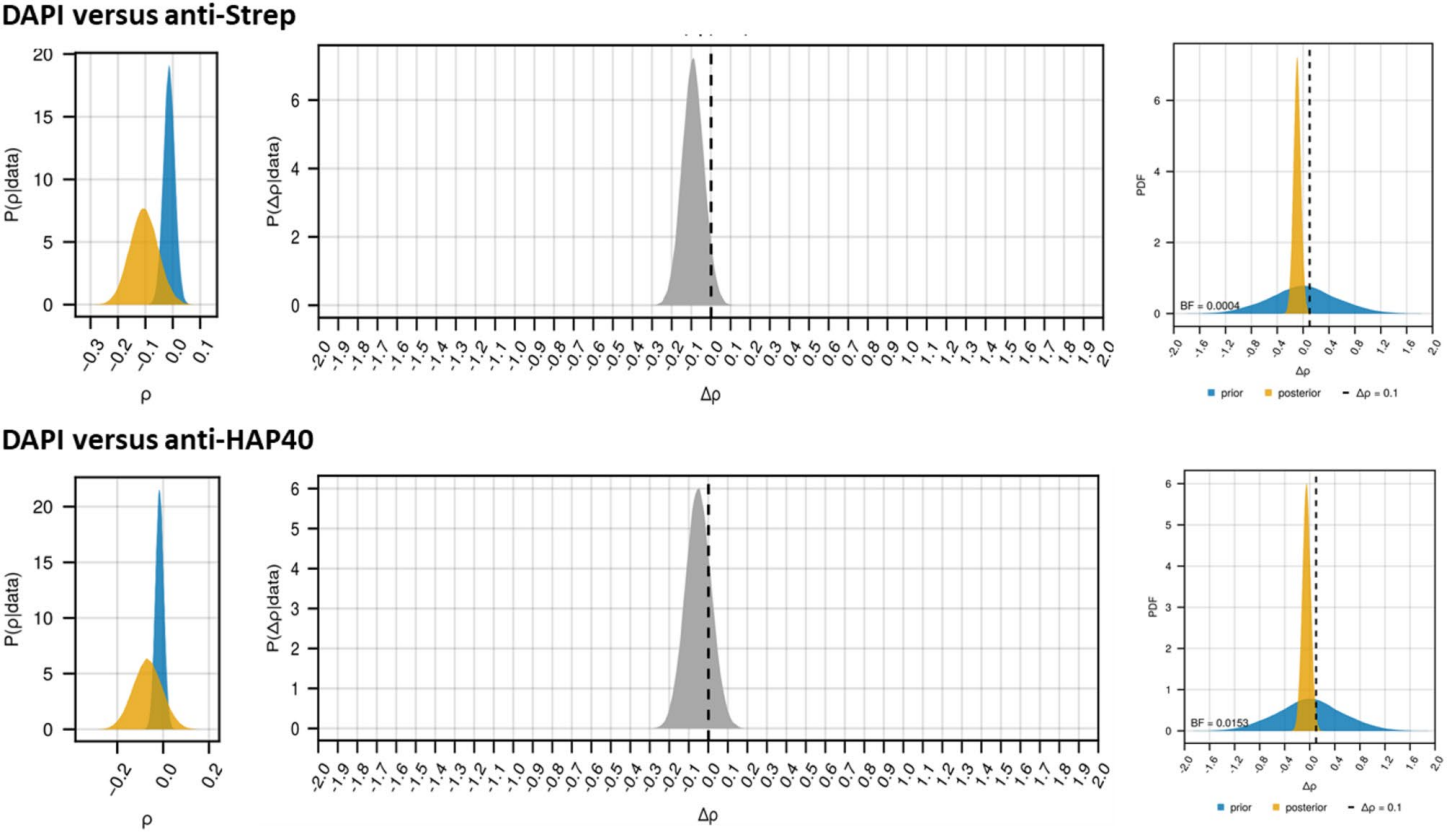
Results of the colocalization analyses between recombinant HAP40 and DAPI. Left: posterior distribution of the Pearson correlation in images from HAP40-Strep transfected cells (orange) and the negative control (blue). Middle: posterior distribution of the difference of Pearson correlations ${\Delta }\rho$ between the images from HAP40-Strep transfected cells and negative control images. Right: prior and posterior distribution of the Pearson correlation in images from HAP40-Strep transfected cells and the Bayes factor $BF\left[ {H_{1} :{\Delta }\rho > 0.1:{ }H_{0} :{\Delta }\rho \le 0.1} \right]$.

Next, we also investigated whether HAP40 signals from either antibody co-localised with DNA using 4′,6-Diamidin-2-phenylindol (DAPI) staining as a marker. With both antibodies, our analysis revealed evidence against a colocalization between HAP40 and DNA (Fig. 3). Bayes factors of 1:2375.30 (anti-Strep antibody) and 1:65.24 (anti-HAP40 antibody) for the alternative hypothesis over the null hypothesis provide extreme or very strong evidence against a significant colocalization (Δρ > 0.1). Additionally, the posterior mean of the colocalization coefficient ρ for HAP40 with DNA were negative in both cases: − 0.105 (95% credible interval [− 0.206; − 0.003]) and − 0.069 (95% credible interval [− 0.193;0.056]) for the anti-Strep antibody and anti-HAP40 antibody, respectively. Hence, although overexpressed HAP40 localises within the cell nucleus, it does not co-localise with DNA.

### Exemplary analysis 2: Colocalization of recombinant HAP40 and HTT in co-transfected A549 cells.

In the second analysis, we explored the usability of ProteinCoLoc to quantify the colocalization between HAP40 and HTT. Given the highly abundant interaction between HAP40 and HTT^10,11^, we expect a clear colocalization between HAP40 and HTT. However, since the labelling of endogenous HAP40 only provides weak signals (data not shown), we decided to overexpress the two proteins. Thereby, the presence of the HTT-HAP40 complex in the studied cells is ensured as such a method was successfully used by us and others to produce and purify the HTT-HAP40 complex and study its structure^10,12,14^. Briefly, we co-transfected the two plasmids pBSK-CMV-HAP40TS (coding for HAP40 recombinantly fused with a c-terminally-fused TwinStrep®-tag)^10^ and the pBSK-CMV-17QHTT (coding for human full-length HTT with a C-terminally fused FLAG®-tag)^15^ into A549 cells using Lipofectamine™ 3000 (Invitrogen™). These two plasmids have been generated and applied earlier in our research^10,15^. The polyclonal anti-HAP40 antibody (Santa Cruz, sc-69489) and an anti-FLAG-tag antibody (Sigma, F3165) were used to detect HAP40 and HTT, respectively. Bayesian analysis was conducted with 10,000 iterations, 100,000 posterior samples, 32 × 32 patches, and a Δρ threshold of 0.1. Control images were generated by shuffling blocks of 3 × 3 pixels.

**Figure 4:**
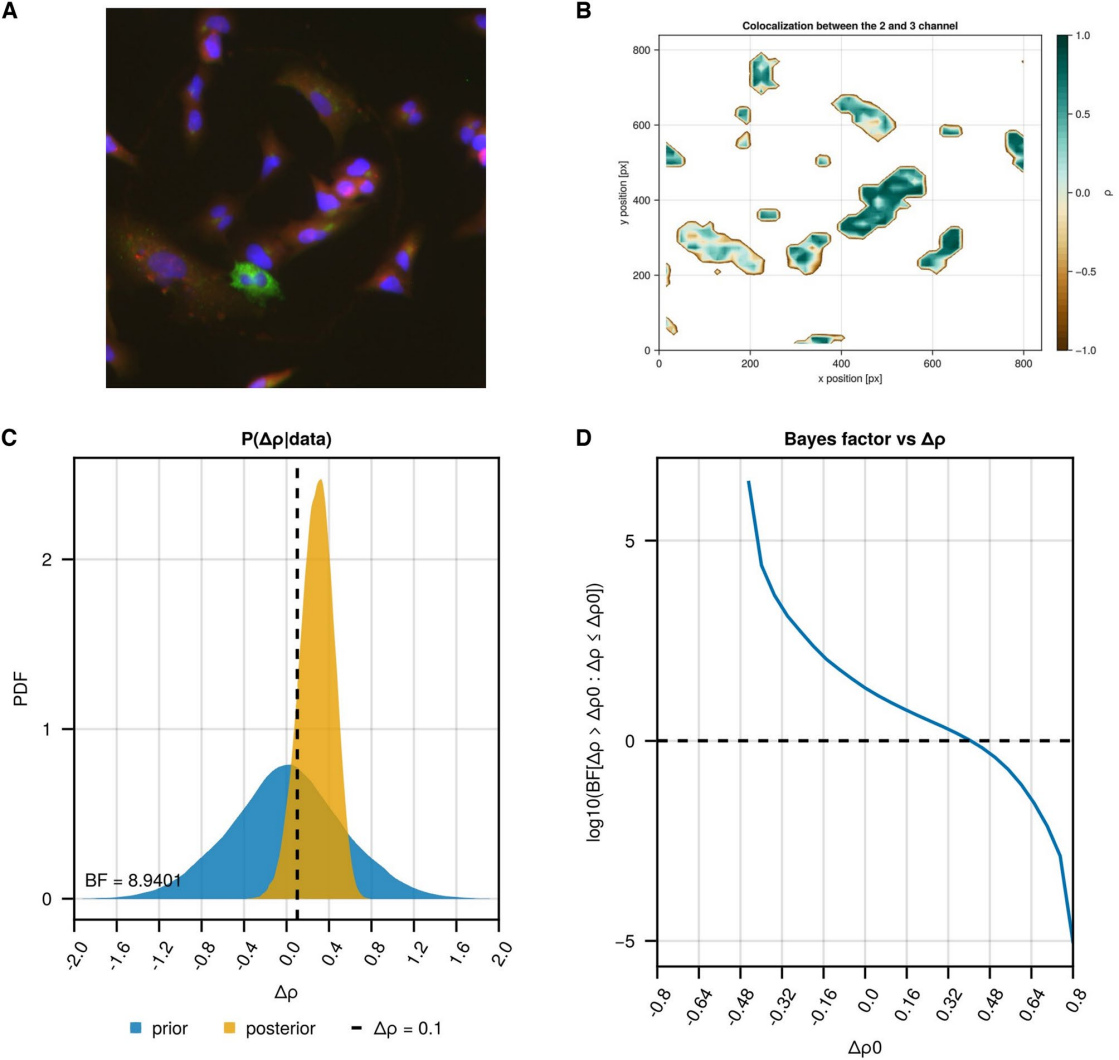
Result of exemplary analysis 2—colocalization between HAP40-Strep and full-length 17QHTT-FLAG HAP40. (**a**) Co-staining of HAP40 with the anti-HAP40 antibody sc-69489 (Donkey anti-goat Alexa Fluor 555, red) and anti-FLAG antibody (Donkey anti-mouse Alexa-Fluor 468). DNA was stained with DAPI and is depicted in blue. (**b**) Local correlation plot between the signal from the anti-HAP40 antibody sc-69489 and anti-Strep antibody. (**c**) Bayes factor plot displaying the prior and posterior distribution for the global ${\Delta }\rho$ between control images (i.e. non-transfected cells, N = 6) and the images from the co-staining (N = 6). (**d**) Bayes factor range plot displaying the Bayes factor at different thresholds for ${\Delta }\rho$.

Using Pearson’s correlation as the correlation metric, the analysis with ProteinCoLoc provided evidence for a colocalization (Δρ > 0.1) between recombinant HAP40 and HTT with a Bayes factor of 8.94:1 for the alternative hypothesis $H_{A} :\Delta \uprho > 0.1$ over the null hypothesis $H_{0} :{\Delta }\rho \le 0.1$ (Fig. 4 and Supplementary file S2). This colocalization occurs mostly in the cytoplasm, as expected from prior results^6^. The mean of the posterior distribution of the Pearson correlation coefficient $\rho$ between the HAP40 and HTT signal was estimated at 0.265 (95% CI [− 0.056; 0.548]), whereas the posterior mean of block-shuffled images was estimated at − 0.014 (95% CI [− 0.014, − 0.027]). Comparable results were obtained using Spearman’s rank correlation to compute the correlation within the patches. Here, the analysis also provided evidence for a colocalization (Δρ > 0.1) between recombinant HAP40 and HTT with a Bayes factor of 8.50:1 and the posterior mean was estimated at 0.278 (95% CI [− 0.048; 0.561]).

These results align well with the expected partial colocalization behaviour of two proteins, A and B, known to form a stable complex in vitro and which have been overexpressed by co-transfection. This method indeed ensures the presence of the A–B complex (accounting for the colocalization events), but also allows A and B to exist independently within the cellular environment. Specially, they may (1) interact exclusively with other cellular partners, such as proteins C and D (e.g., A–C, B–D), (2) they may have different expression levels (e.g., due to a difference in transfection efficiency), or (3) they may experience protein folding issues upon overexpression preventing their proper interaction.

## Discussion

Here, we described the development and implementation of ProteinCoLoc—a Julia package and compiled standalone software^21^ for the quantitative analysis of colocalization between proteins or other fluorescently tagged molecules using microscopic images. Our software allows the automation of image analysis, making it suitable for high-throughput projects, and addresses several limitations inherent to current methodologies by integrating robust Bayesian statistical models, thereby enhancing the accuracy and reliability of colocalization assessments.

One of the critical features of ProteinCoLoc is its ability to effectively distinguish between signal and background noise. Traditional methods often struggle with this differentiation leading to skewed colocalization metrics. However, by utilising Otsu’s thresholding^5^, ProteinCoLoc minimises these inaccuracies providing a more precise analysis of protein colocalization. This advancement will be particularly beneficial in high-throughput settings, where manual adjustments are impractical.

The adoption of a Bayesian hierarchical model in ProteinCoLoc offers several advantages in the analysis of protein colocalization. Its ability to model complex data structures and incorporate various sources of uncertainty significantly enhances the robustness and reliability of colocalization estimates. Hierarchical models excel at handling the multi-level nature of biological data, crucial for differentiating within-sample variability from between-sample variability^22,23^, and accurately interpreting spatial relationships across different cells or conditions. However, implementing the Bayesian hierarchical model does prolong computation times potentially limiting the speed and scalability of analysis. Currently, the loading of images and Bayesian inference for a dataset of six images per group, three colour channels and 16 patches per image takes approximately 200s on a machine with an AMD Ryzen5 2600 processor (Supplementary file S5). In general, the time and space complexity of ProteinCoLoc (Bayesian inference) scales quadratically with the number of images and with the number of patches (Supplementary file S5). Plotting operations scale linearly with the image count, generating 3n + 3 plots per dataset. Despite these computational demands, given ProteinCoLoc’s autonomous operation post-initiation and the significant increase in robustness by accounting for both within-sample and between-sample variability, the extended computation time is justified by the benefits of the Bayesian approach, ensuring more accurate and reliable colocalization analysis.

Furthermore, ProteinCoLoc’s implementation of local correlation analysis allows for a more nuanced understanding of spatial relationships. This feature is invaluable when studying complex cellular environments, where global analyses might overlook localised interactions. By focusing on specific regions within the cellular milieu, researchers can obtain a detailed understanding of protein distributions and interactions.

The applicability and utility of ProteinCoLoc were demonstrated through two exemplary analyses focusing on the colocalization of HAP40 with different antibodies and its colocalization with HTT. Studying the colocalization of HAP40 with HTT is of interest to understand the pathophysiology of Huntington disease^8^, a neurodegenerative disorder characterised by the abnormal aggregation of the HTT protein^24^. While these studies showcase the software’s capabilities, it is crucial to note that the quality of any colocalization analysis is inherently dependent on the quality of the input data^2,3,25^. Thus, it remains essential for users to ensure optimal imaging conditions and proper experimental design. Without proper controls minimising imaging artifacts and artefactual signals due to an unspecific binding of antibodies, ProteinCoLoc’s result will be inaccurate. Therefore, we highly recommend the optimisation of experimental conditions (e.g., to optimise the signal-to-noise ratio and to reduce pixel oversaturation) as described earlier^2^. For example, a low signal-to-noise ratio and high background signals may interfere with the results. Although ProteinCoLoc incorporates Otsu’s thresholding for background detection, differentiating signal from noise may be inaccurate, particularly in specimens with low contrast or uneven fluorescence. Second, we recommend the use of reference images such as samples without secondary antibodies. These reference images can be used to account for autofluorescence and artefactual signals due unspecific binding of the secondary antibodies. Nonetheless, the implemented Bayesian hierarchical model employing Student t-distributions to model the correlation metrices is more robust to outliers due to their heavier tails in comparison to a normal distribution and hence mitigates the effects of outliers (e.g. patches with exceptionally high correlation values). Furthermore, a high dispersion of the posterior distributions and high posterior means for the inter-image σ and intra-image variability $\sigma_{n} , \ldots , \sigma_{n}$ with n images (Fig. 2) may indicate the presence of imaging artefacts or other data-related issues.

## Conclusion

In conclusion, ProteinCoLoc is a fully validated, user-friendly, robust, and versatile tool for the quantitative analysis of protein colocalization. By leveraging the power of Bayesian hierarchical models, it addresses the limitations of traditional colocalization techniques, offering improved accuracy and the ability to handle complex data.

ProteinCoLoc’s innovative approach to handling both global and local colocalization assessments, coupled with its user-friendly interface, will make it an invaluable tool for researchers studying complex protein interactions in cellular environments. The inclusion of a graphical user interface and a compiled standalone application^21^ should facilitate widespread adoption by researchers in various fields. As an open-source project ProteinCoLoc also invites continual development and adaptation further enhancing its impact and utility for the scientific community.

## Methods

ProteinCoLoc has been entirely implemented in Julia v. 1.10^26^ (https://julialang.org/) and is accessible both as a Julia package, available at https://github.com/ma-seefelder/ProteinCoLoc, and as a standalone, platform-independent, and compiled application, accessible via the following 10.5281/zenodo.10977960^21^. The software, along with its source code, is released under the GNU Affero General Public License v3.0 (https://www.gnu.org/licenses/agpl-3.0.en.html), permitting its usage for both non-commercial and commercial applications. All dependencies for the Julia package are documented in the Project.toml and Manifest.toml files, and the package can be effortlessly installed, inclusive of all necessary packages, using Julia’s built-in Package Manager. Bayesian inference, sampling and model generation functionalities are executed using Turing.jl^27^. Plot generation uses GLMakie.jl^28^ and the GUI was implemented with Mousetrap.jl^29^. Notably, no additional dependencies are required for the compiled version of ProteinCoLoc. The Julia package, ProteinCoLoc.jl, is particularly well-suited for analysing a high number of conditions and images in high-throughput settings.

### Input images and naming convention

For downstream analysis with ProteinCoLoc, it is ideal to capture images together with the respective control images (e.g. secondary-antibody-only controls) with identical settings, like exposure times, gains, and laser intensities. Facilitating analysis without reference image, we have incorporated two alternative options for generating control images through augmentation of the provided input images. Firstly, the “pixel-wise shuffling” method randomly shuffles the pixels in each image channel. Secondly, following the proposal by Dunn et al.^3^, a “block-wise shuffling” scheme is available, where small blocks of 3 × 3 pixels are shuffled to retain the influence of autocorrelation.

ProteinCoLoc can load images with various file types (tiff, tif, jpg, jpeg, png). To enable the successful loading of images into ProteinCoLoc all images belonging to the same biological condition should be in the same folder and the individual channels should be named as $\left[ {\text{image name}} \right]\_{\text{c}}\left[ {\text{channel ID}} \right].{\text{tiff}}$. The $\left[ {image name} \right]$ can also comprise “_” if the last underscore is followed by ${\text{c}}\left[ {channel ID} \right]$ where the channel ID lies between 1 and the number of recorded channels. For example, 20231212_HEK293_HAP40_c1.tiff might be an acceptable file name for the sample 20231212_HEK293_HAP40 and channel 1 feinCoLoc imposes no limitations on for ProteinCoLoc.

ProteinCoLoc imposes no limitations on the number of recorded channels, image dimensions or number of images. Unlike the compiled version, the Julia package ProteinCoLoc.jl package comprises the function convert_lif_to_tiff(path) that can convert LIF files into tiff-files following the aforementioned naming convention.

### Background detection using Otsu’s thresholding.

Background detection using Otsu’s thresholding is a critical step in our methodology. Otsu’s thresholding is a well-established image-processing technique introduced by Nobuyuki Otsu^5^. This method optimally determines the threshold that minimises the intra-class variance of pixel intensities, effectively segmenting the image into foreground (signal) and background components^5^. To elaborate, Otsu’s thresholding computes the optimum threshold by maximising the variance between two classes of pixels, assuming a bimodal distribution of pixel intensities^5^. Considering the background noise in the analysis would negatively impact the results of any colocalization analysis, as it would reduce the observed colocalization values. In protein colocalization analysis, this technique aids in automatically discerning and isolating the background signal, which is crucial for accurate quantification of colocalization between two fluorescently labelled entities. Furthermore, automatic background detection reduces the dependency on user intervention and enhances the reproducibility of the colocalization analysis.

### Local correlation

Understanding the spatial correlation patterns within cellular regions is crucial for gaining detailed insights into the distribution of proteins. Global correlation analysis may overlook localised interactions, limiting our ability to comprehend specific aspects of cellular processes. Local correlation analysis aims to address this limitation by focusing on the correlation patterns in defined small patches of the input images. To facilitate the identification of spatial/local colocalization patterns, two different outputs can be generated.

First, the patched correlation plot in ProteinCoLoc provides a visual representation of the correlation strength between two proteins or molecular probes across discrete patches or regions within an image. The number of image patches can be defined by the user. Each patch corresponds to a defined area of the image, allowing researchers to identify localised variations in colocalization. This plot aids in pinpointing specific cellular regions where proteins exhibit distinct correlation patterns, contributing to a more nuanced understanding of their spatial relationships. Second, ProteinCoLoc introduces the local correlation plot (Fig. 1B), which goes beyond global assessments and allows for the visualisation of local colocalization patterns at a finer spatial scale than the patched correlation plot. This plot enables visualising variations in colocalization intensity within small image patches (ideally between 10 and 100 px^2^) and helps in uncovering nuanced spatial relationships, for example, in certain subcellular compartments. Robust estimates for individual patches are ensured by performing a correlation between intensities only if 15 or more pixels in a patch have intensities above Otsu’s threshold for both channels. Where the defined patch number is too high, the software dynamically adjusts, attempting different patch numbers until a suitable configuration is identified. This adaptive approach ensures the robust computation of local correlation even in complex scenarios and with large datasets where a manual adjustment of patch numbers remains infeasible.

### Bayesian analysis

In the Bayesian analysis, we first compute the correlation of pixel intensities between channels A and B across image patches using Pearson’s^30^, Spearman’s rank^31^, or Kendall’s rank correlation^31^ coefficients. The Pearson’s correlation coefficient assesses linear relationships, whereas Spearman’s and Kendall’s are rank-based methods that detect non-linear dependencies^31^. Though Spearman’s and Kendall’s correlation coefficients are highly similar, Spearman’s correlation is particularly advantageous when many tied ranks are present, offering more accurate results due to its average ranking method^32^.

**Figure 5:**
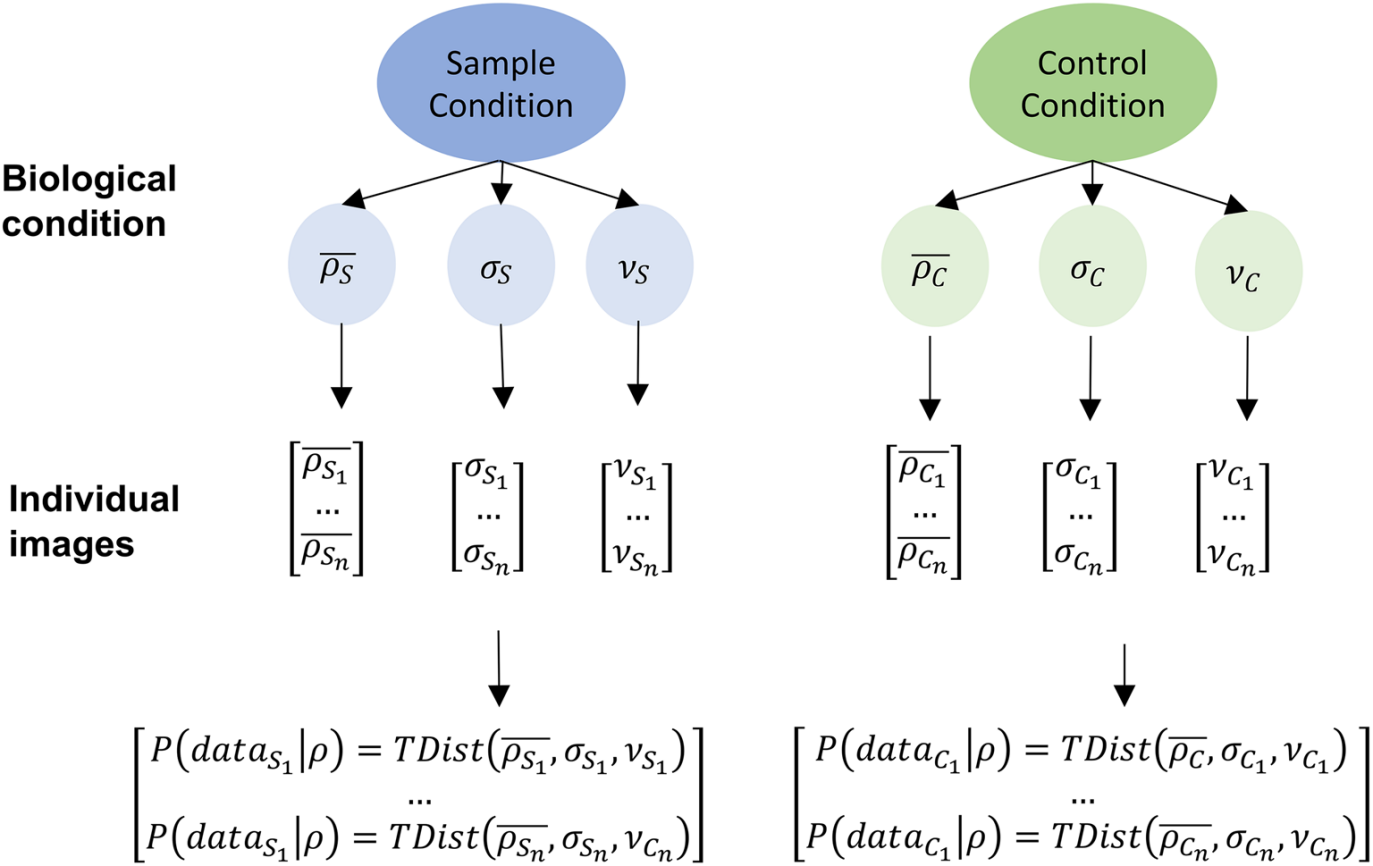
Bayesian hierarchical model for protein colocalization. This diagram illustrates the Bayesian hierarchical model employed in ProteinCoLoc to assess the correlation between pixel intensities within patches of image I. The likelihood is calculated using a Student T-distribution parametrised by the mean correlation coefficient $\overline{{\rho_{I} }}$, the dispersion parameter $\sigma_{I}$ and the degrees of freedom $\nu_{I}$. These parameters for individual images are sampled from global hyperparameters $\rho_{S}$, $\sigma_{S}$, and $\nu_{S}$ for images within the sample condition or from the global hyperparameters $\rho_{C}$, $\sigma_{C}$, and $\nu_{C}$ for images from the control condition.

Next, we employ a two-level hierarchical model, as depicted in Fig. 5. This model utilises a Student T-distribution to calculate the likelihood of a correlation between pixel intensities within patches of an image. The key parameters include the mean correlation coefficient $\overline{{\rho_{I} }}$, dispersion parameter $\sigma_{I}$, and degrees of freedom $\nu_{I}$. These parameters for individual images are sampled from global hyperparameters $\rho_{S}$, $\sigma_{S}$, and $\nu_{S}$ in the case of images within the sample condition, or $\rho_{C}$, $\sigma_{C}$, and $\nu_{C}$ for images within the control condition. The specifications of priors and hyperpriors can be found in Table 1. This hierarchical approach enhances the robustness and reliability of the colocalization analysis while acknowledging the parameter uncertainty stemming from the variability within and between captured images. To reduce the computation time and resource requirements, we employ Variational Inference (VI) with Automatic Differentiation Variational Inference (ADVI) to approximate the posterior distributions as implemented in the Julia package Turing.jl^27^.

**Table 1:** Prior specification of hierarchical Bayesian model.

Level	Parameter	Prior
Biological condition	$\overline{{{\varvec{\rho}}_{{\varvec{S}}} }} ,\user2{ }\overline{{{\varvec{\rho}}_{{\varvec{C}}} }}$	$\overline{{\rho_{S} }} \sim Cauchy\left( {0,0.3} \right) = \frac{1}{{0.3\pi \left( {1 + \left( {\frac{x}{0.3}} \right)^{2} } \right)}}, \overline{{\rho_{S} }} \in \left( { - 1,1} \right)$ $\overline{{\rho_{C} }} \sim Cauchy\left( {0,0.3} \right) = \frac{1}{{0.3\pi \left( {1 + \left( {\frac{x}{0.3}} \right)^{2} } \right)}}, \overline{{\rho_{C} }} \in \left( { - 1,1} \right)$
	${\varvec{\sigma}}_{{\varvec{S}}} ,{\varvec{\sigma}}_{{\varvec{C}}}$	$\sigma_{S} \sim Cauchy\left( {0,0.3} \right) = \frac{1}{{0.3\pi \left( {1 + \left( {\frac{x}{0.3}} \right)^{2} } \right)}}, \sigma_{S} \in \left( {0,1} \right)$ $\sigma_{C} \sim Cauchy\left( {0,0.3} \right) = \frac{1}{{0.3\pi \left( {1 + \left( {\frac{x}{0.3}} \right)^{2} } \right)}}, \sigma_{C} \in \left( {0,1} \right)$
	${\varvec{\nu}}_{{\varvec{S}}} ,\user2{ \nu }_{{\varvec{C}}}$	$\nu_{S} \sim Exponential\left( {\theta = 1} \right) = e^{ - x}$ $\nu_{C} \sim Exponential\left( {\theta = 1} \right) = e^{ - x}$
	${\varvec{\tau}}_{{\varvec{S}}} ,{\varvec{\tau}}_{{\varvec{C}}}$	$\tau_{S} \sim Cauchy\left( {0,0.3} \right) = \frac{1}{{0.3\pi \left( {1 + \left( {\frac{x}{0.3}} \right)^{2} } \right)}}, \sigma_{S} \in \left( {0,1} \right)$ $\tau_{C} \sim Cauchy\left( {0,0.3} \right) = \frac{1}{{0.3\pi \left( {1 + \left( {\frac{x}{0.3}} \right)^{2} } \right)}}, \sigma_{C} \in \left( {0,1} \right)$
Image level	$\overline{{{\varvec{\rho}}_{{{\varvec{S}}_{{\varvec{I}}} }} }} ,\user2{ }\overline{{{\varvec{\rho}}_{{{\varvec{C}}_{{\varvec{I}}} }} }}$	$\overline{{\rho_{{S_{I} }} }} \sim Normal\left( {\overline{{\rho_{S} }} ,\sigma_{S} } \right) \in \left( { - 1,1} \right)$ $\overline{{\rho_{{C_{I} }} }} \sim Normal\left( {\overline{{\rho_{C} }} ,\sigma_{C} } \right) \in \left( { - 1,1} \right)$
	${\varvec{\sigma}}_{{{\varvec{S}}_{{\varvec{I}}} }} ,{\varvec{\sigma}}_{{{\varvec{C}}_{{\varvec{I}}} }}$	$\sigma_{{S_{I} }} \sim Normal\left( {\sigma_{S} ,\tau_{S} } \right) \in \left( {0,1} \right)$ $\sigma_{{C_{I} }} \sim Normal\left( {\sigma_{C} ,\tau_{C} } \right) \in \left( {0,1} \right)$
	${\varvec{\nu}}_{{{\varvec{S}}_{{\varvec{I}}} }} ,\user2{ \nu }_{{{\varvec{CI}}}}$	$\nu_{SI} \sim Exponential\left( {\theta = \nu_{S} } \right) = \frac{1}{{\nu_{S} }} \cdot e^{{ - \left( {\frac{x}{{\nu_{S} }}} \right)}}$ $\nu_{SC} \sim Exponential\left( {\theta = \nu_{S} } \right) = \frac{1}{{\nu_{C} }} \cdot e^{{ - \left( {\frac{x}{{\nu_{C} }}} \right)}}$

**Figure 6:**
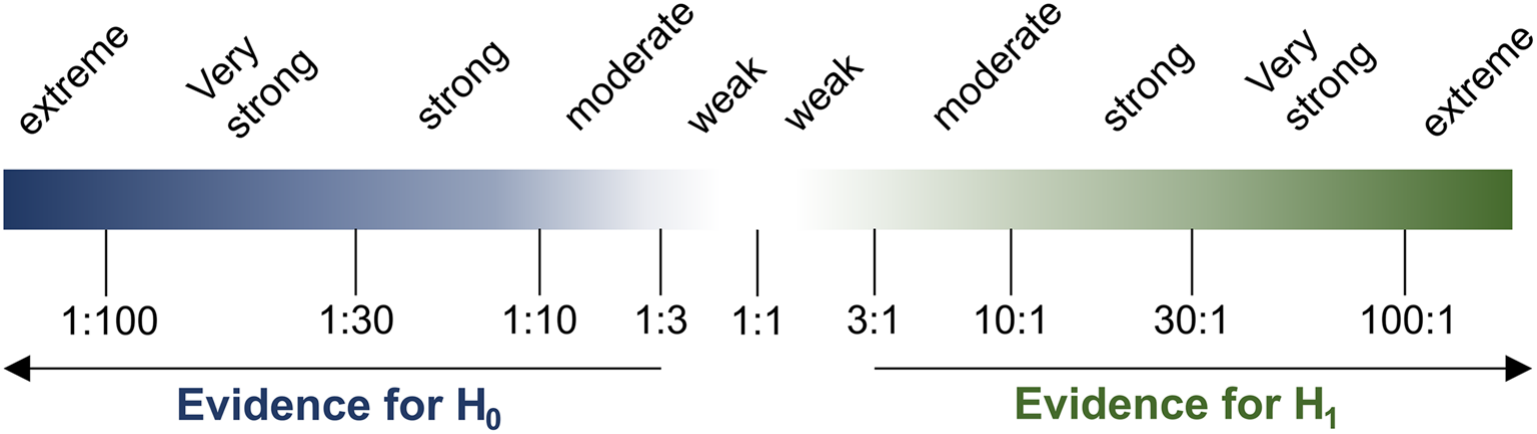
Strength of evidence of obtained Bayes factors. This figure illustrates the frequently applied interpretation scale after Lee and Wagenmakers^33^. The Bayes factors are shown as the BF[H_1_:H_0_] displaying the relative likelihood of H_1_ to H_0_.

The result of the Bayesian analysis can be interpreted with the help of three generated plots. First, a posterior plot displays the posterior distribution of all global parameters, namely $\rho , \nu , \sigma , \tau$, and ${\Delta }\rho$, for both biological conditions (referred to as “posterior plot” in the GUI) (Fig. 2). Second, a plot of the prior and posterior distribution for the difference in the mean correlation ${\Delta }\rho = \overline{{\rho_{S} }} - \overline{{\rho_{C} }}$ and the respective Bayes factor (BF)

$$BF\left[ {H_{1} :\Delta \rho > \Delta \rho_{0} : H_{0} :\Delta \rho \le \Delta \rho_{0} } \right] = \frac{{P\left( {\Delta \rho > \Delta \rho_{0} |data} \right)}}{{P\left( {\Delta \rho \le \Delta \rho_{0} |data} \right)}} \cdot \frac{{P\left( {\Delta \rho \le \Delta \rho_{0} } \right)}}{{P\left( {\Delta \rho > \Delta \rho_{0} } \right)}}$$

with a user-specified threshold ${\Delta }\rho_{0}$ can be calculated (Fig. 1C). The BF quantifies the likelihood ratio between the competing hypotheses $H_{1} :{\Delta }\rho > {\Delta }\rho_{0}$ over $H_{0} :{\Delta }\rho \le {\Delta }\rho_{0}$. Hence, a BF of n indicates that the data provides n-times more evidence for H_1_ than H_2_. Unlike frequentist null hypothesis testing, the BF provides evidence for both hypotheses rather than solely against the null hypothesis. Two common discrete interpretation scales for Bayes factors are shown in Fig. 6^33^.

As a small difference in colocalization is biologically difficult to interpret, a default value of ${\Delta }\rho_{0} = 0.1$ is utilised, but users may opt for higher values for a more stringent test criterion. As the Bayes factor is sensitive to the selected ${\Delta }\rho_{0}$, a third plot, the Bayes factor range plot, displays the relationship between the decadic logarithm of the Bayes Factor and the threshold ${\Delta }\rho_{0}$ (Fig. 1D).

### Benchmarking

Benchmarking of the code was performed using the @time macro from Julia’s standard library^26,34^. For benchmarking a machine with an AMD Ryzen5 2600 and 32 GB RAM (2166 MHz) were used. The benchmarking script is available in ProteinCoLoc’s GitHub repository (https://github.com/ma-seefelder/ProteinCoLoc).

### Cell culture and expression of recombinant HAP40 and HTT

A549 cells, originating from adenocarcinoma human alveolar basal epithelial tissues, isolated from a 58-year-old male patient’s explanted tumour^35^, were cultured in Minimal Essential Media (Gibco) supplemented with 10% v/v fetal bovine serum (Gibco) and 1% v/v Penicillin–Streptomycin-Glutamine (Gibco).

For the expression of recombinant HAP40 and HTT, cells were transfected utilizing Lipofectamine 3000 in accordance with the manufacturer’s guidelines, with 0.5 µg of DNA per plasmid. Specifically, human full-length HAP40 with a C-terminal Twin-Strep tag was encoded by a pBSK-CMV derived plasmid^10,15^ and full-length human HTT with a C-terminal FLAG-tag^10^ was similarly encoded and transfected into A549 cells. As negative control, A549 cells were transfected with the empty pBSK-CMV plasmid, which does not express any transgene.

### Immunocytochemistry

48 h post transfection, samples were fixed using 4% formaldehyde solution after extensive washing with DPBS. Given the intracellular expression of HAP40 and HTT, permeabilization of fixed A549 cells was achieved with 0.1% saponin in DPBS and non-specific antibody binding was minimized using a blocking solution containing (2% bovine serum albumin, 5% fetal bovine serum, and 1% saponin). Thereafter, cells were incubated with primary antibodies detecting HAP40 (Santa Cruz, sc-69489), the TwinStrep tag on HAP40 (IBA, 2–1507-001), or the FLAG-tag (Sigma, F3165) on the recombinantly expressed HTT. As secondary antibodies, Alexa 488 or Alexa 546 conjugated donkey anti-goat, anti-rabbit, or anti-mouse antibodies were employed (Invitrogen) and DNA was stained using 0.1 µg/ml 4′,6-diamidino-2-phenylindole (DAPI). To preserve fluorescence, cells were mounted using Prolong Diamond Antifade mountant and images were collected with the Leica TCS SP8 confocal laser scanning microscope.

### Supplementary Information

Supplementary Legends.

Supplementary Information 1.

Supplementary Information 2.

Supplementary Information 3.

Supplementary Information 4.

Supplementary Information 5.

## Data Availability

All wet-lab data generated or analysed during this study are included in this published article and its supplementary files. The Julia source code can be accessed at https://github.com/ma-seefelder/ProteinCoLoc^21^. Furthermore, the standalone compiled version of ProteinCoLoc can be accessed via the following 10.5281/zenodo.10977960.
