# Supplementary material for: ProteinCoLoc streamlines Bayesian analysis of colocalization in microscopic images
Source: Sci Rep. 2024 Jun 10;14:13277. doi: 10.1038/s41598-024-63884-1 (PMC11164984; doi:10.1038/s41598-024-63884-1)
Supplement: Supplementary file 1 — Supplementary Legends. [file 41598_2024_63884_MOESM1_ESM.docx]

**Supplementary file S1: Co-immunofluorescence images for exemplary analysis 1.** This zip-archive (.zip) contains raw images obtained by laser-scanning confocal microscopy of A549 cells transfected with a HAP40 expression plasmid featuring a c-terminally fused Twin-Strep-tag (“HAP40-TS”), alongside images from A549 cells transfected with an empty plasmid serving as a control (“control”). The archive includes DAPI (channel 1), anti-Strep antibody (channel 2), and anti-HAP40 (Santa Cruz, sc-69489) (channel 3) images.

**Supplementary file S2: Co-immunofluorescence images for exemplary analysis 2.** Co-immunofluorescence Images for Exemplary Analysis 2. This zip archive encompasses raw confocal microscopy images of A549 cells co-transfected with plasmids for expression of HAP40, featuring a c-terminally fused Twin-Strep-tag, and 17Q-HTT, with a c-terminally fused FLAG-tag. The channels included are DAPI (channel 1), anti-HAP40 (Santa Cruz, sc-69489, channel 2), and anti-FLAG antibody (channel 3).

**Supplementary file S3:** Jupyter Notebook with a code example. A comprehensive Jupyter notebook that provides a detailed walkthrough of example analysis, showcasing the application of ProteinCoLoc in a practical setting.

**Supplementary file S4: Step-by-step guide.** This guide provides instructions on utilizing ProteinCoLoc’s graphical user interface (GUI).

**Supplementary file S5: Time and space complexity.**
