# Supplementary figures and images for: ProteinCoLoc streamlines Bayesian analysis of colocalization in microscopic images
Source: Sci Rep. 2024 Jun 10;14:13277. doi: 10.1038/s41598-024-63884-1 (PMC11164984; doi:10.1038/s41598-024-63884-1)

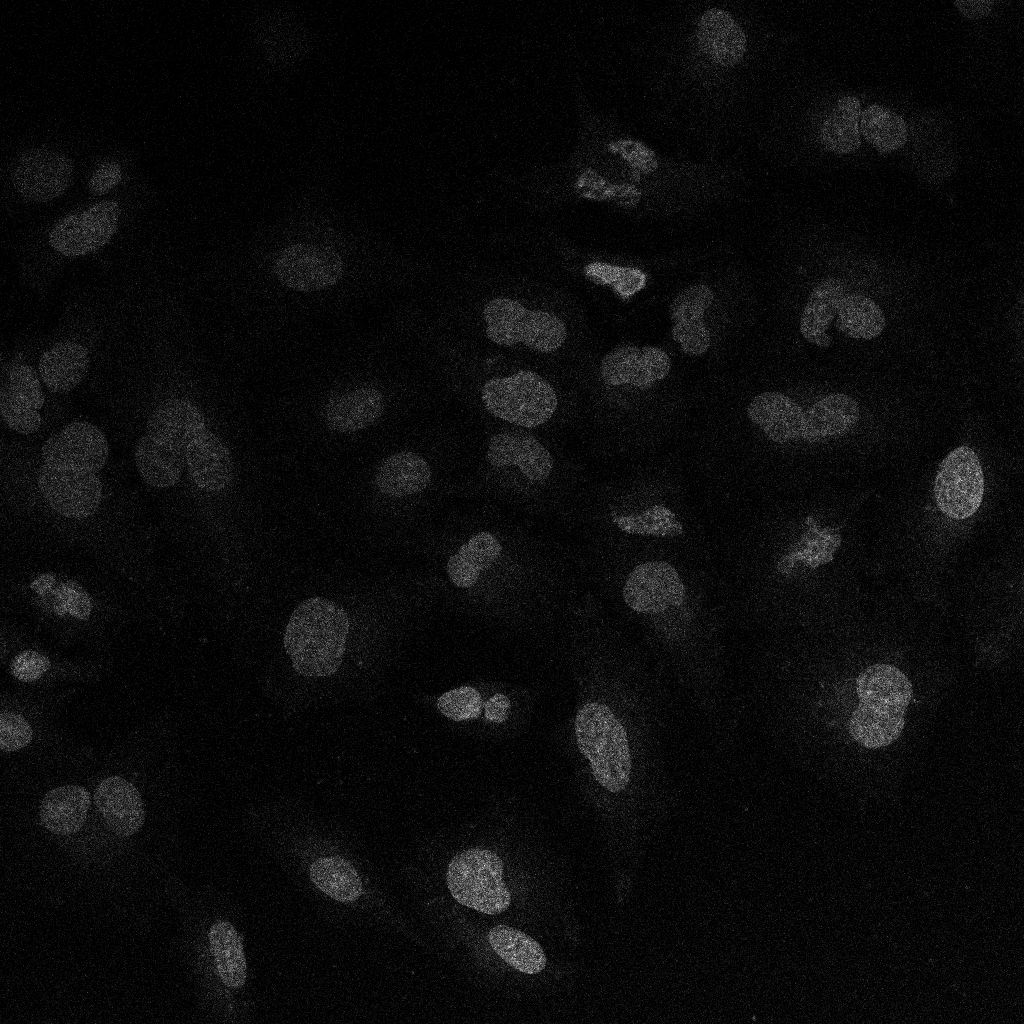

Supplement: Supplementary file 2 — Supplementary Information 1. [file 41598_2024_63884_MOESM2_ESM.zip › Additional_file_1/Control/ctrl_series 002_c1.tif]

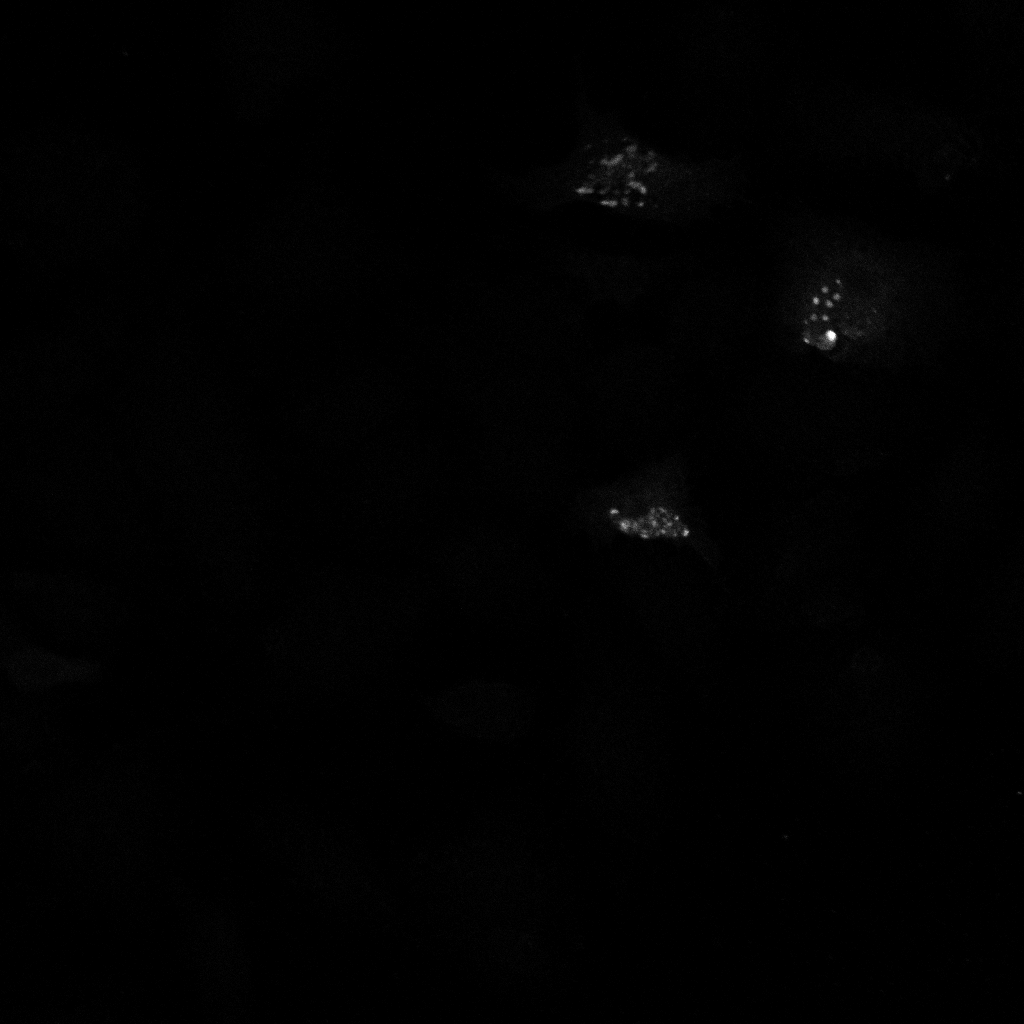

Supplement: Supplementary file 2 — Supplementary Information 1. [file 41598_2024_63884_MOESM2_ESM.zip › Additional_file_1/Control/ctrl_series 002_c2.tif]

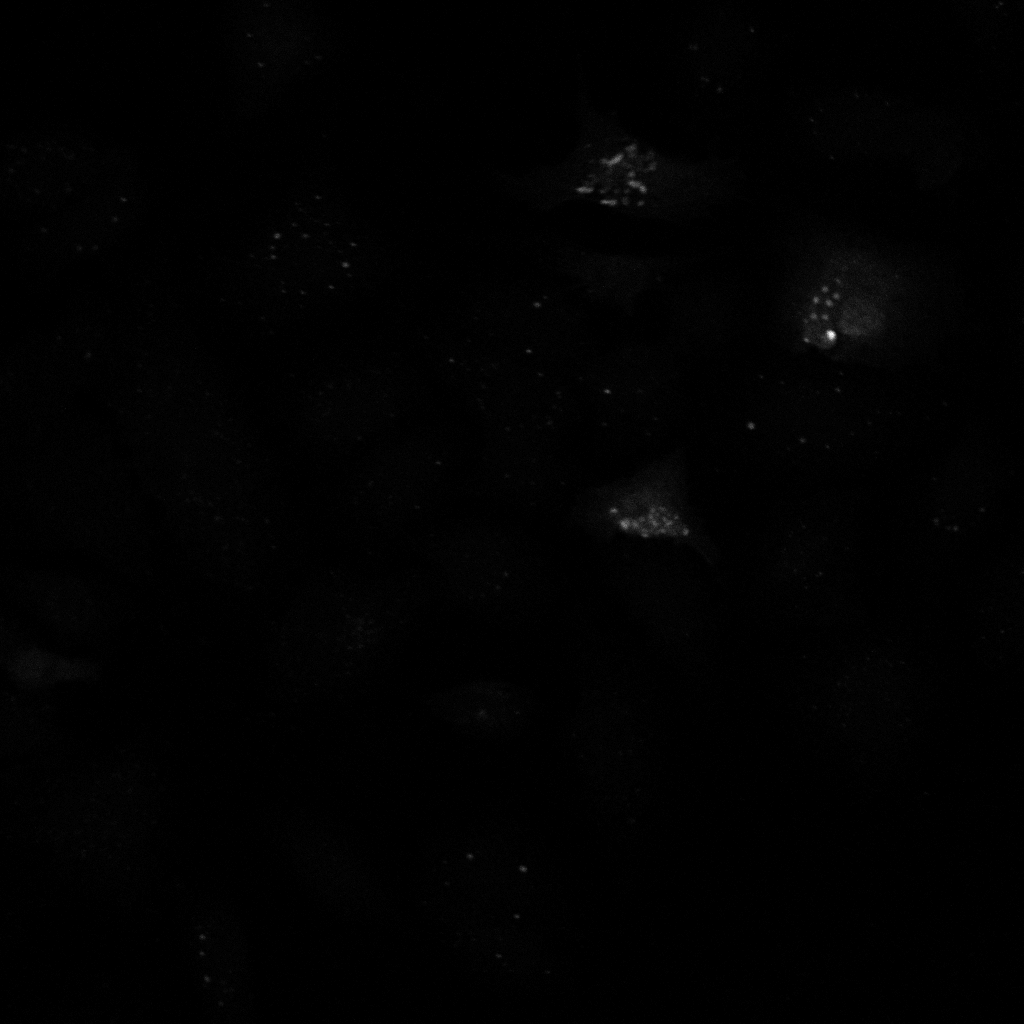

Supplement: Supplementary file 2 — Supplementary Information 1. [file 41598_2024_63884_MOESM2_ESM.zip › Additional_file_1/Control/ctrl_series 002_c3.tif]

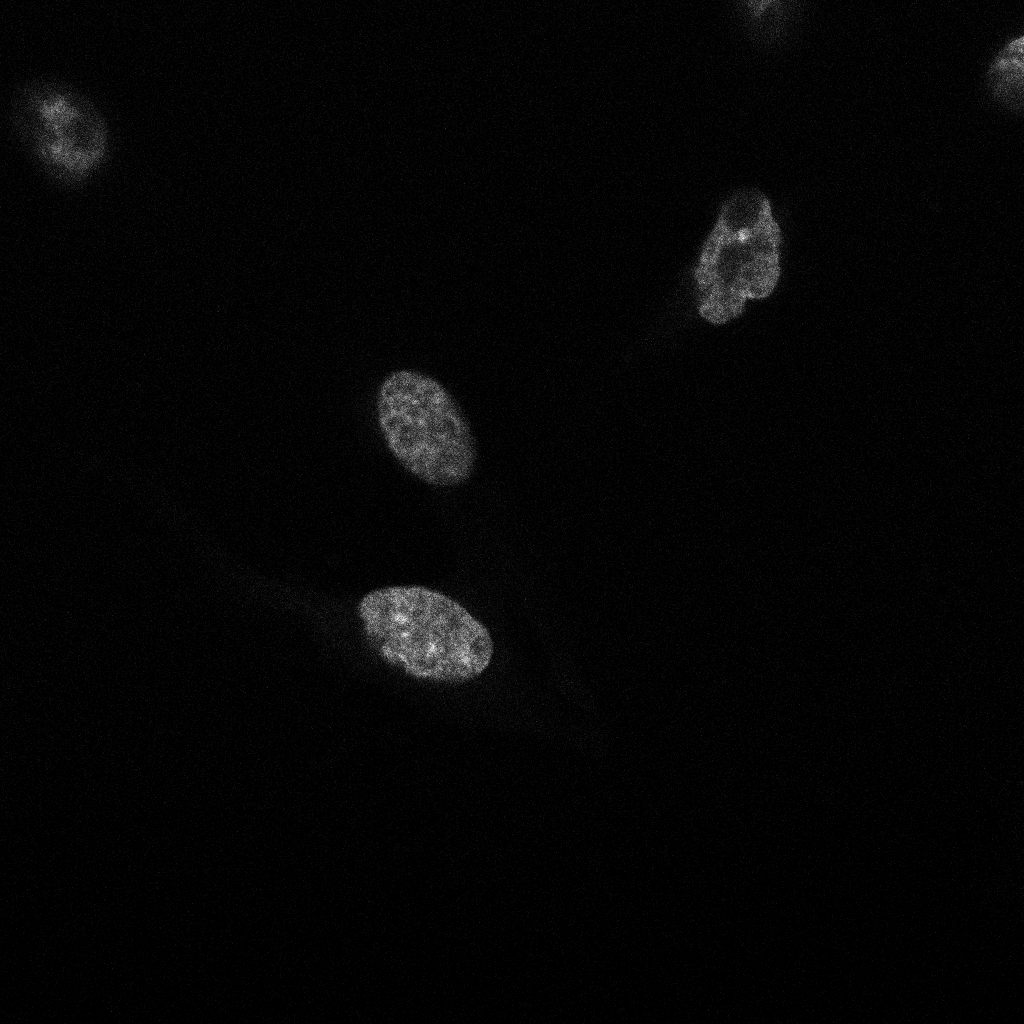

Supplement: Supplementary file 2 — Supplementary Information 1. [file 41598_2024_63884_MOESM2_ESM.zip › Additional_file_1/Control/ctrl_series 006_c1.tif]

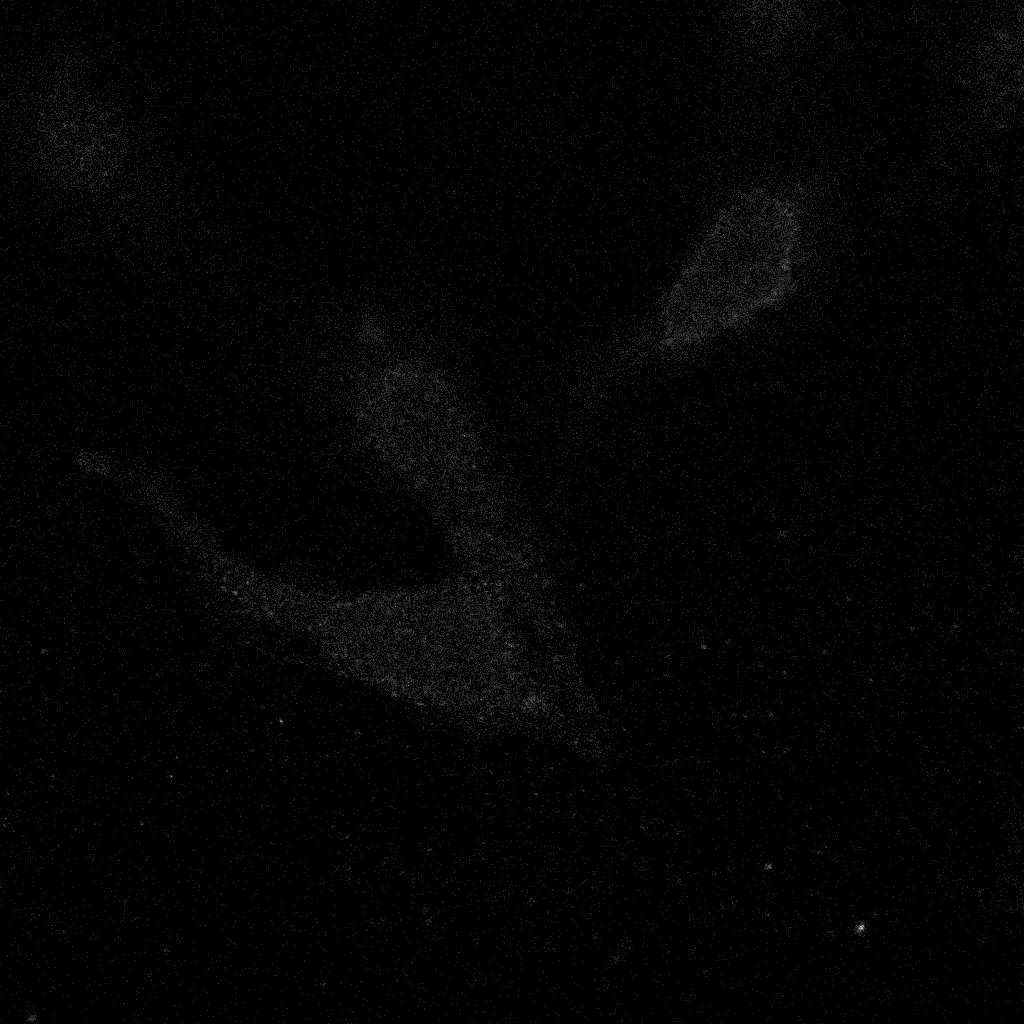

Supplement: Supplementary file 2 — Supplementary Information 1. [file 41598_2024_63884_MOESM2_ESM.zip › Additional_file_1/Control/ctrl_series 006_c2.tif]

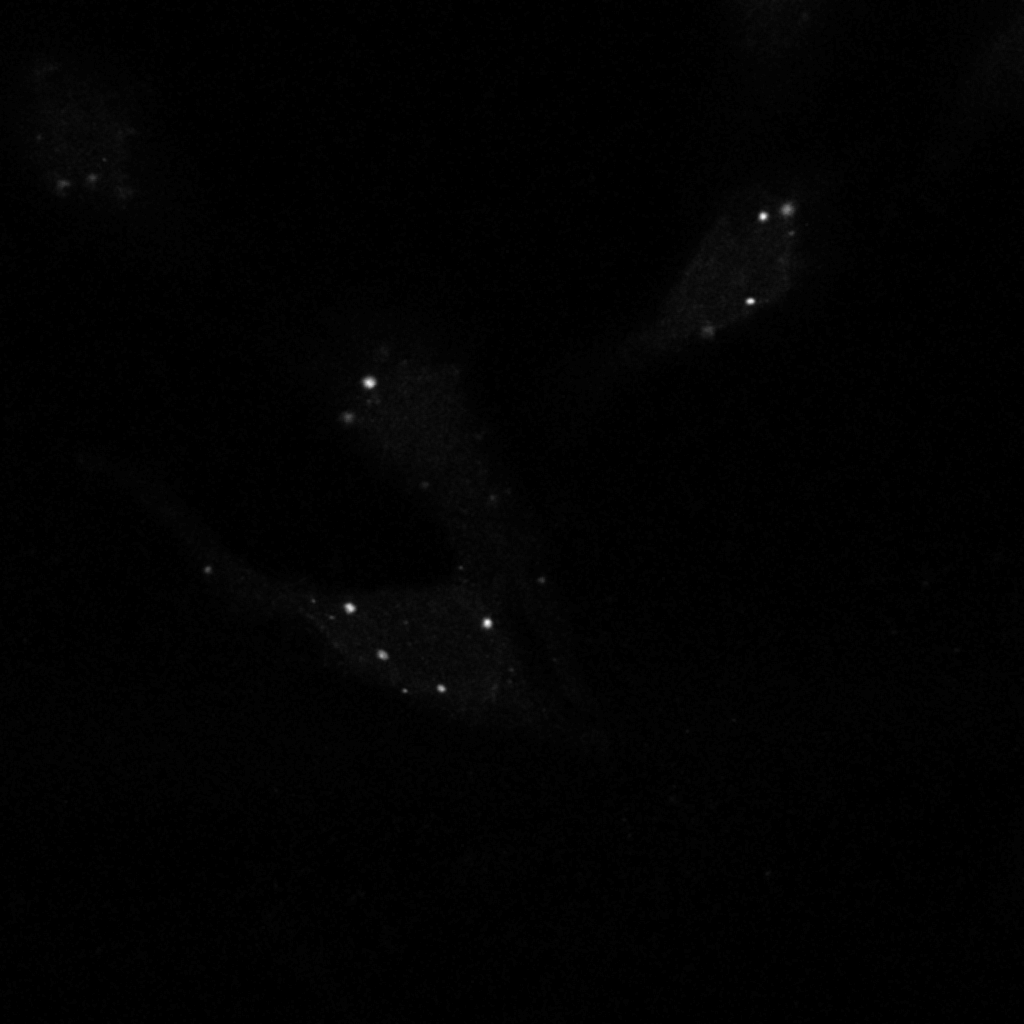

Supplement: Supplementary file 2 — Supplementary Information 1. [file 41598_2024_63884_MOESM2_ESM.zip › Additional_file_1/Control/ctrl_series 006_c3.tif]

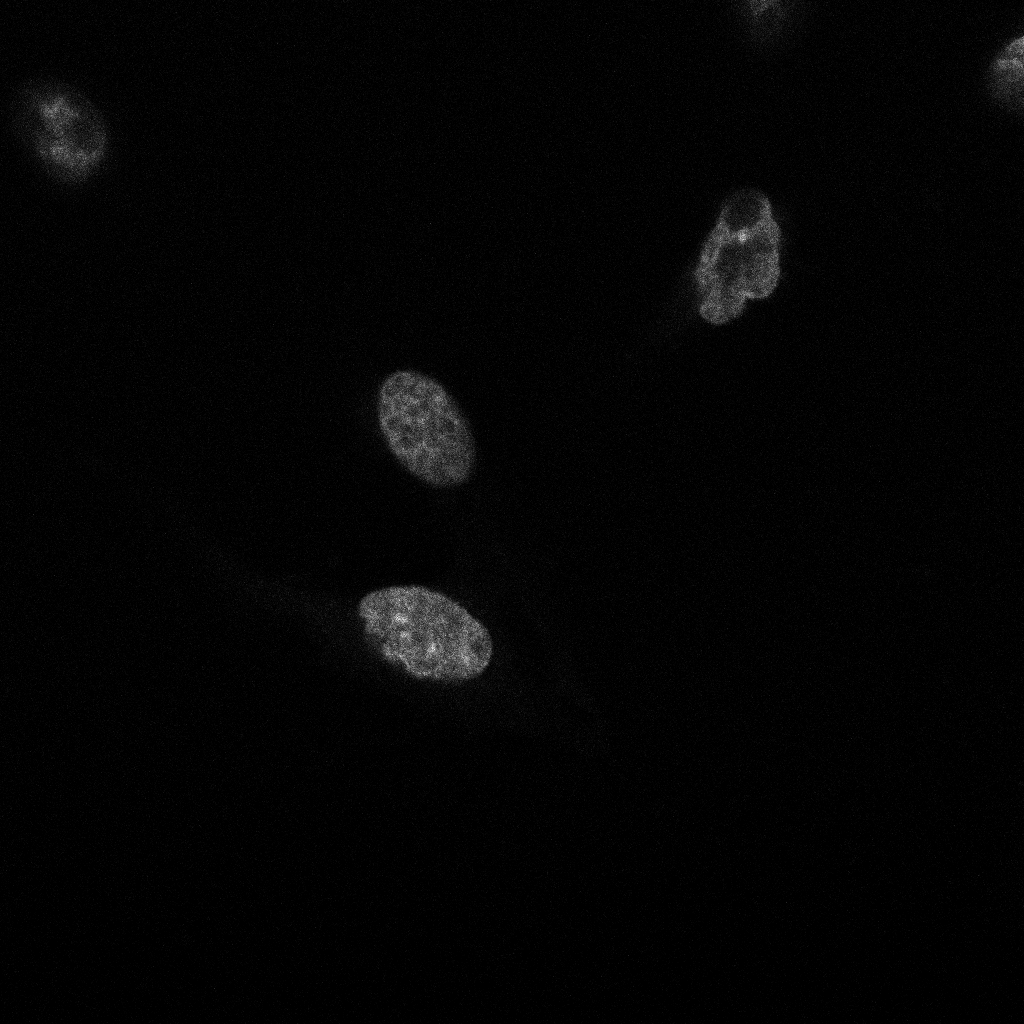

Supplement: Supplementary file 2 — Supplementary Information 1. [file 41598_2024_63884_MOESM2_ESM.zip › Additional_file_1/Control/ctrl_series 008_c1.tif]

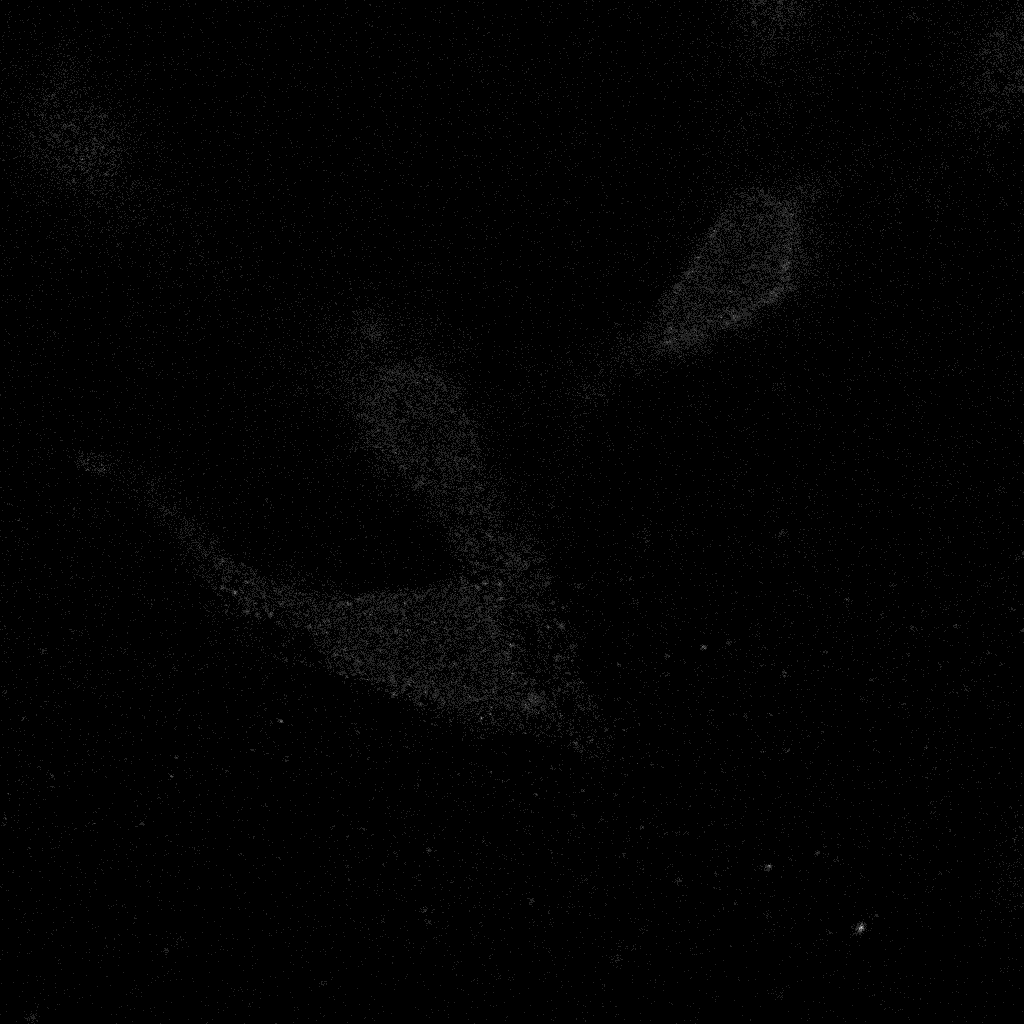

Supplement: Supplementary file 2 — Supplementary Information 1. [file 41598_2024_63884_MOESM2_ESM.zip › Additional_file_1/Control/ctrl_series 008_c2.tif]

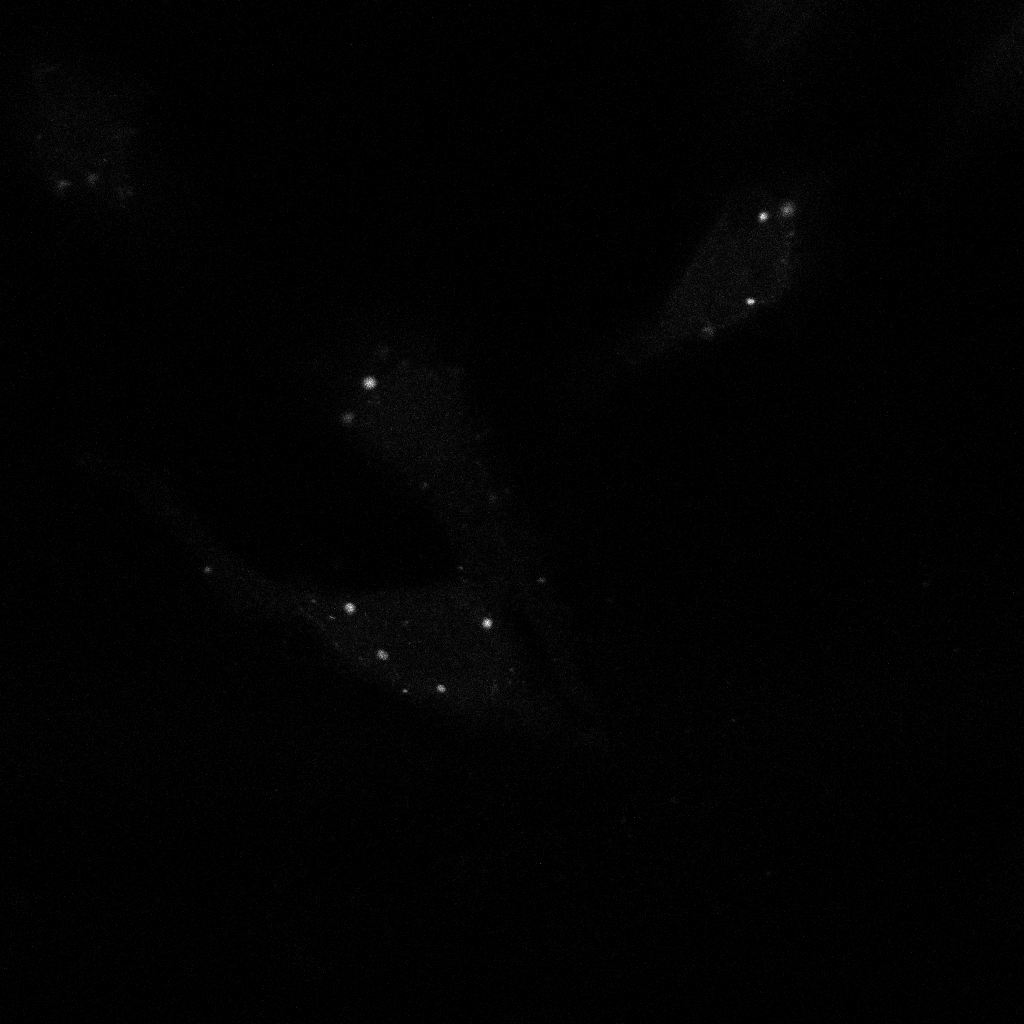

Supplement: Supplementary file 2 — Supplementary Information 1. [file 41598_2024_63884_MOESM2_ESM.zip › Additional_file_1/Control/ctrl_series 008_c3.tif]

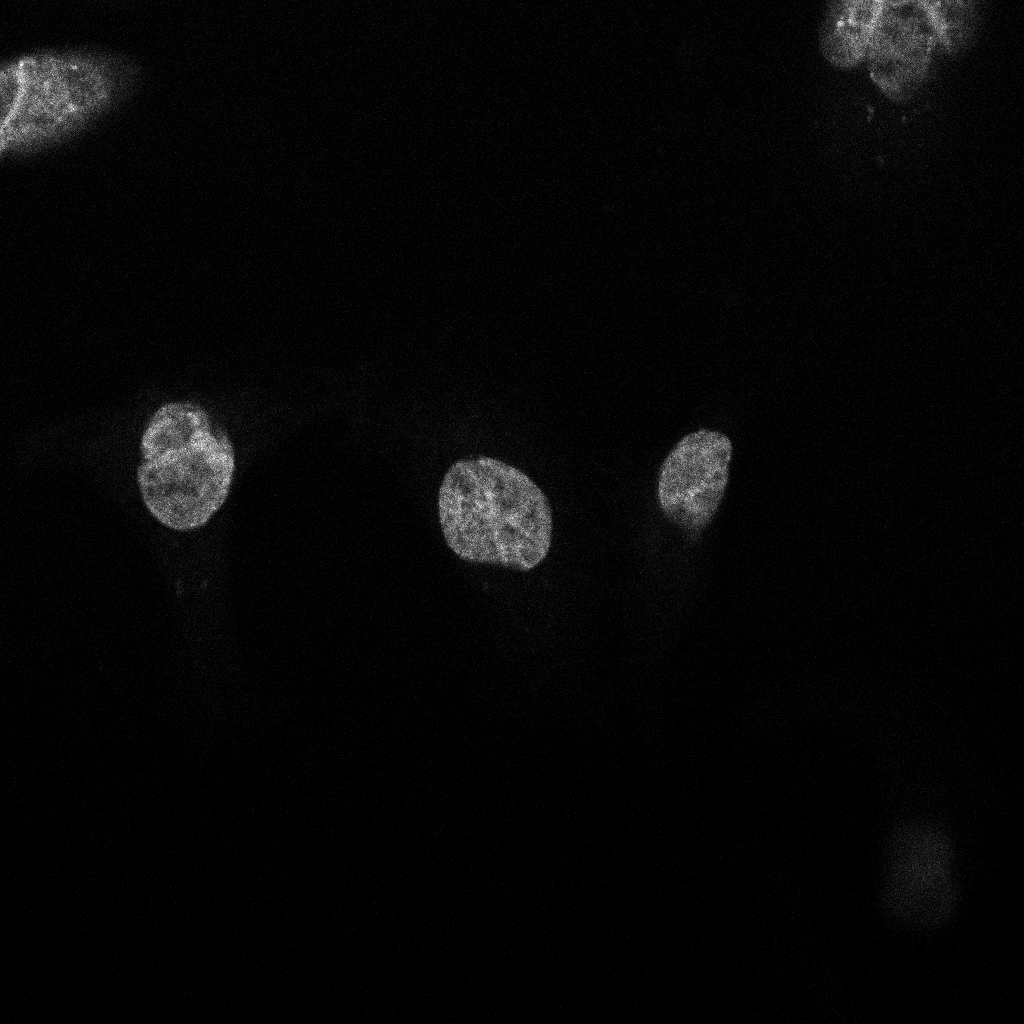

Supplement: Supplementary file 2 — Supplementary Information 1. [file 41598_2024_63884_MOESM2_ESM.zip › Additional_file_1/Control/ctrl_series 010_c1.tif]

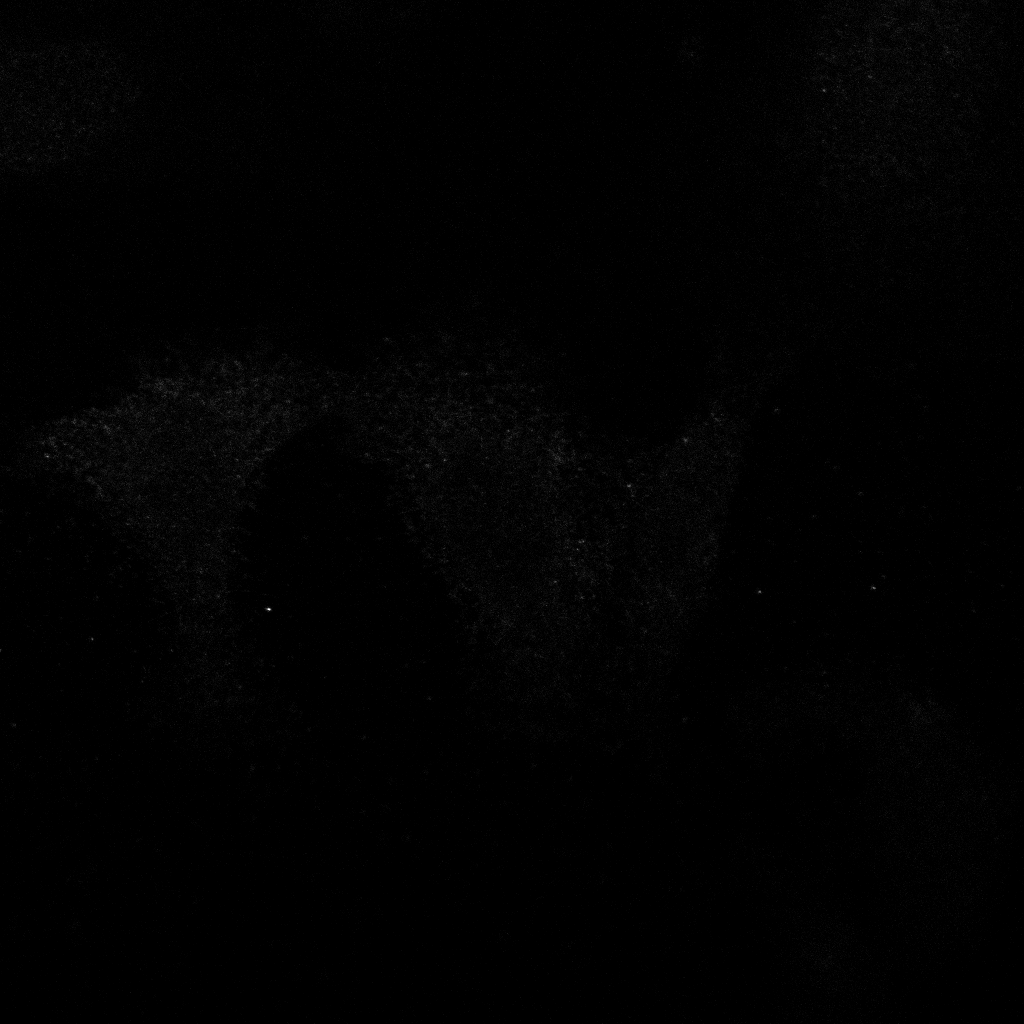

Supplement: Supplementary file 2 — Supplementary Information 1. [file 41598_2024_63884_MOESM2_ESM.zip › Additional_file_1/Control/ctrl_series 010_c2.tif]

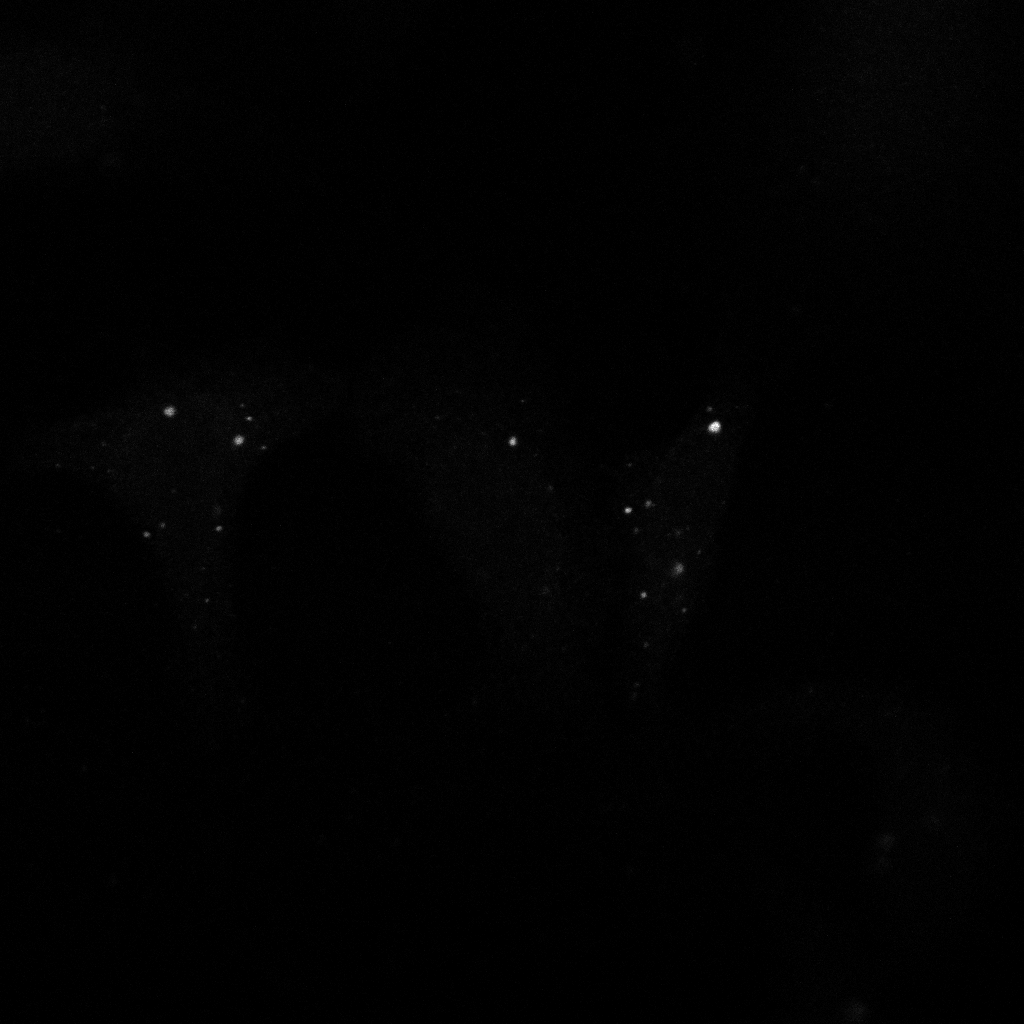

Supplement: Supplementary file 2 — Supplementary Information 1. [file 41598_2024_63884_MOESM2_ESM.zip › Additional_file_1/Control/ctrl_series 010_c3.tif]

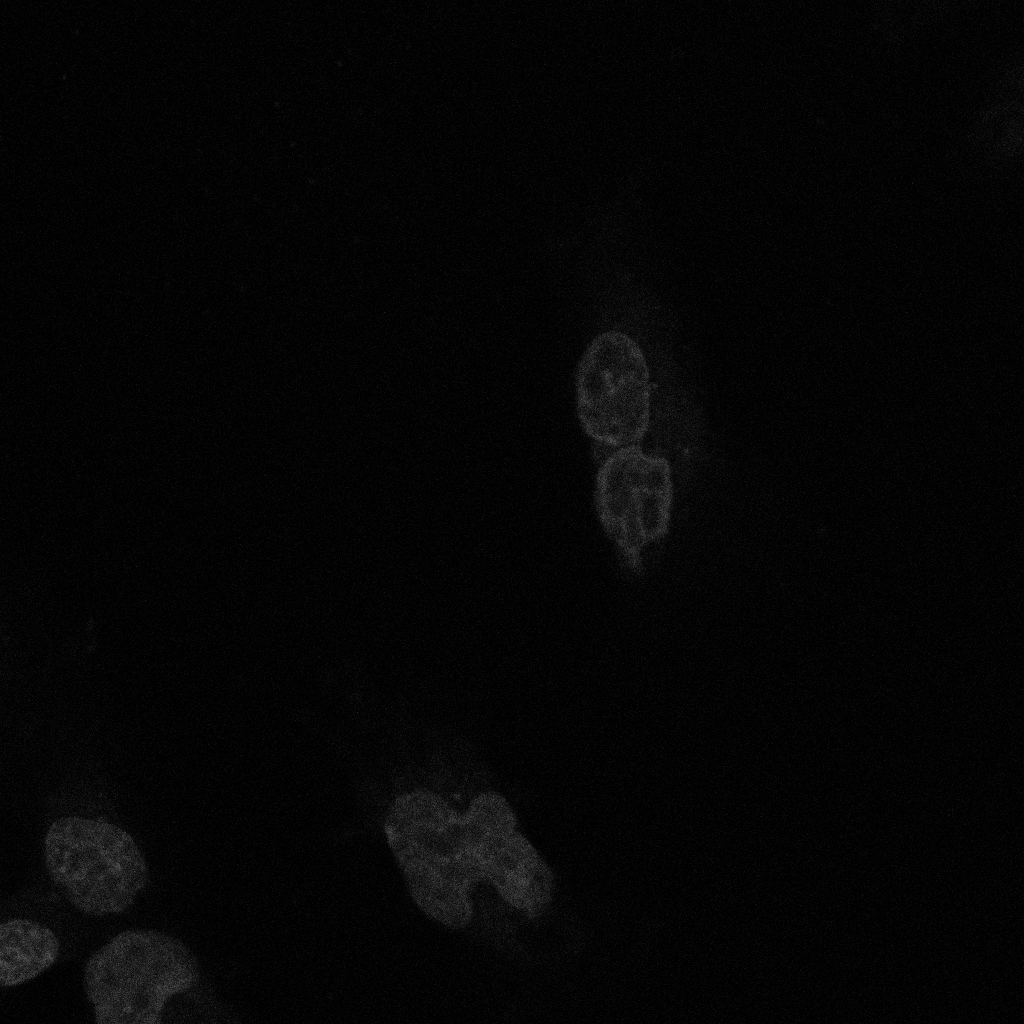

Supplement: Supplementary file 2 — Supplementary Information 1. [file 41598_2024_63884_MOESM2_ESM.zip › Additional_file_1/HAP40-TS/series 0018_c1.tif]

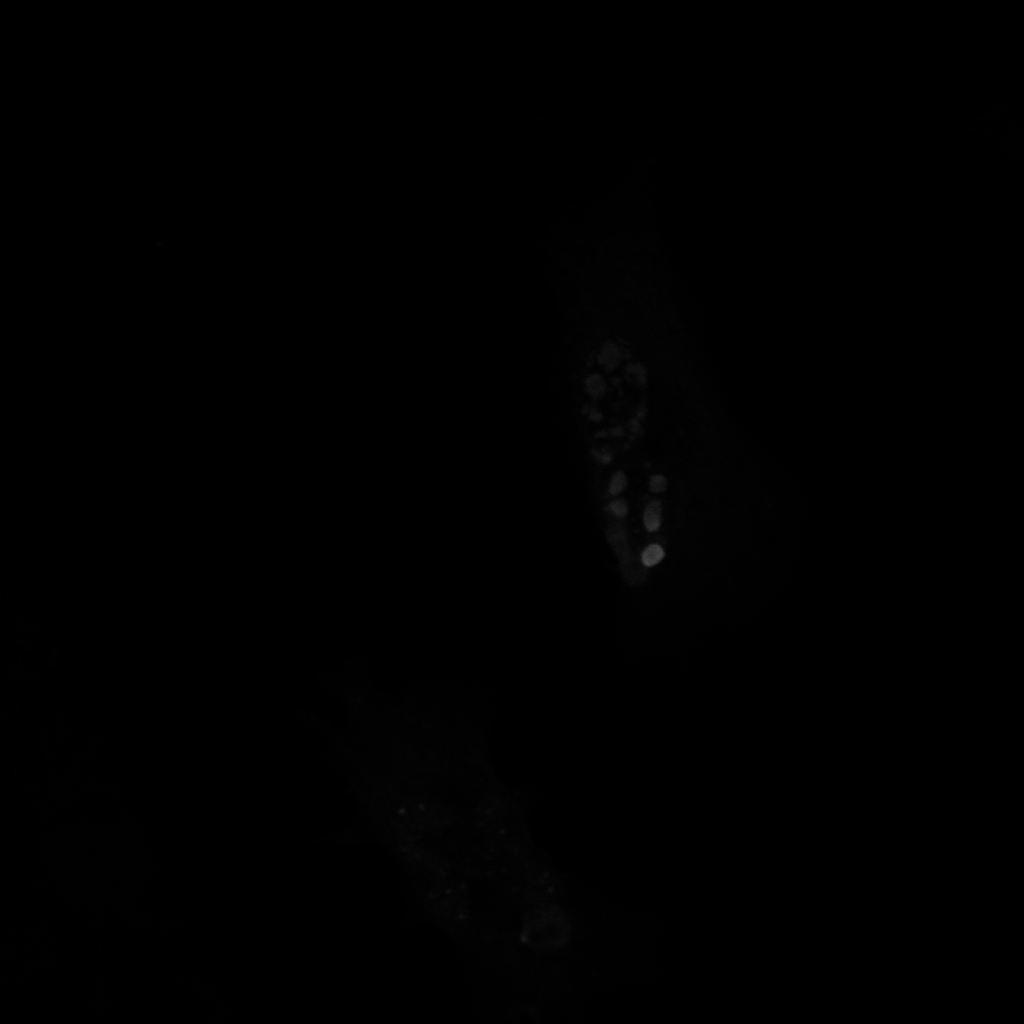

Supplement: Supplementary file 2 — Supplementary Information 1. [file 41598_2024_63884_MOESM2_ESM.zip › Additional_file_1/HAP40-TS/series 0018_c2.tif]

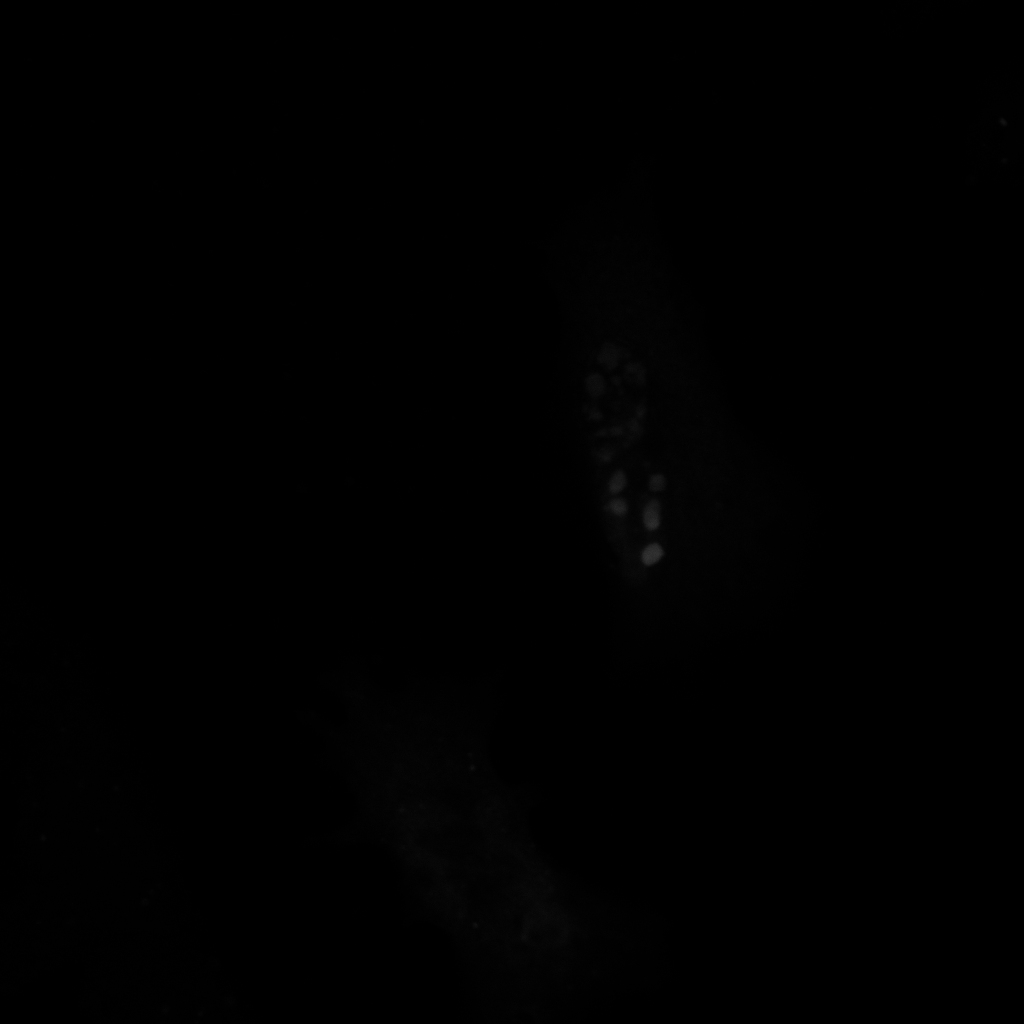

Supplement: Supplementary file 2 — Supplementary Information 1. [file 41598_2024_63884_MOESM2_ESM.zip › Additional_file_1/HAP40-TS/series 0018_c3.tif]

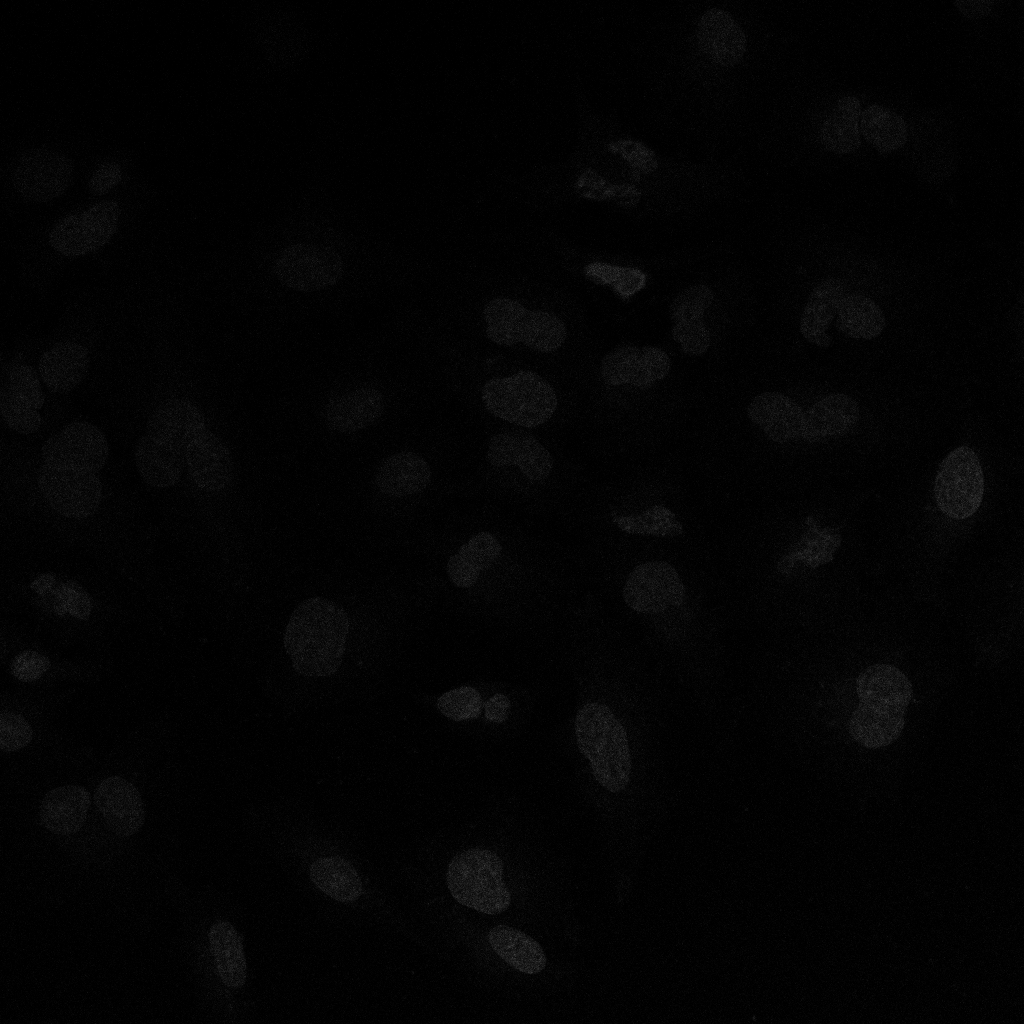

Supplement: Supplementary file 2 — Supplementary Information 1. [file 41598_2024_63884_MOESM2_ESM.zip › Additional_file_1/HAP40-TS/series 002_c1.tif]

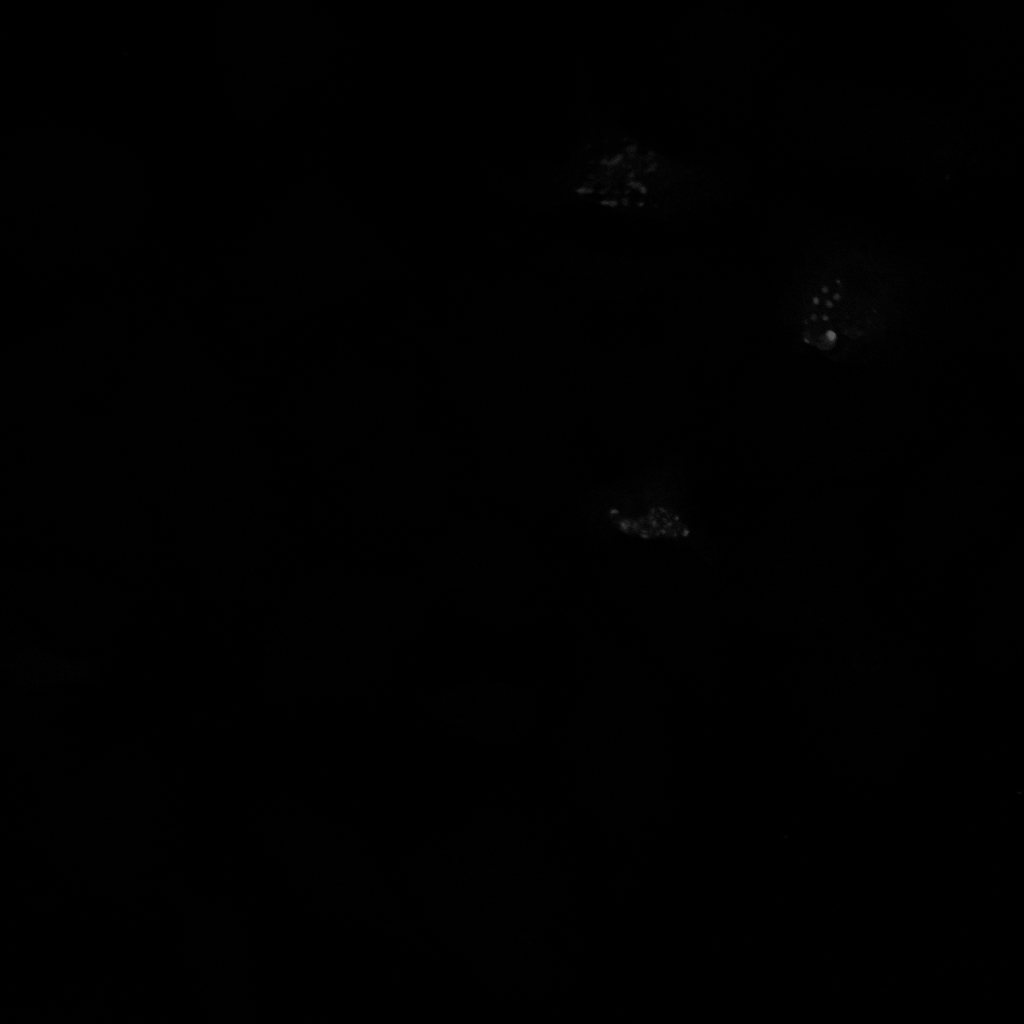

Supplement: Supplementary file 2 — Supplementary Information 1. [file 41598_2024_63884_MOESM2_ESM.zip › Additional_file_1/HAP40-TS/series 002_c2.tif]

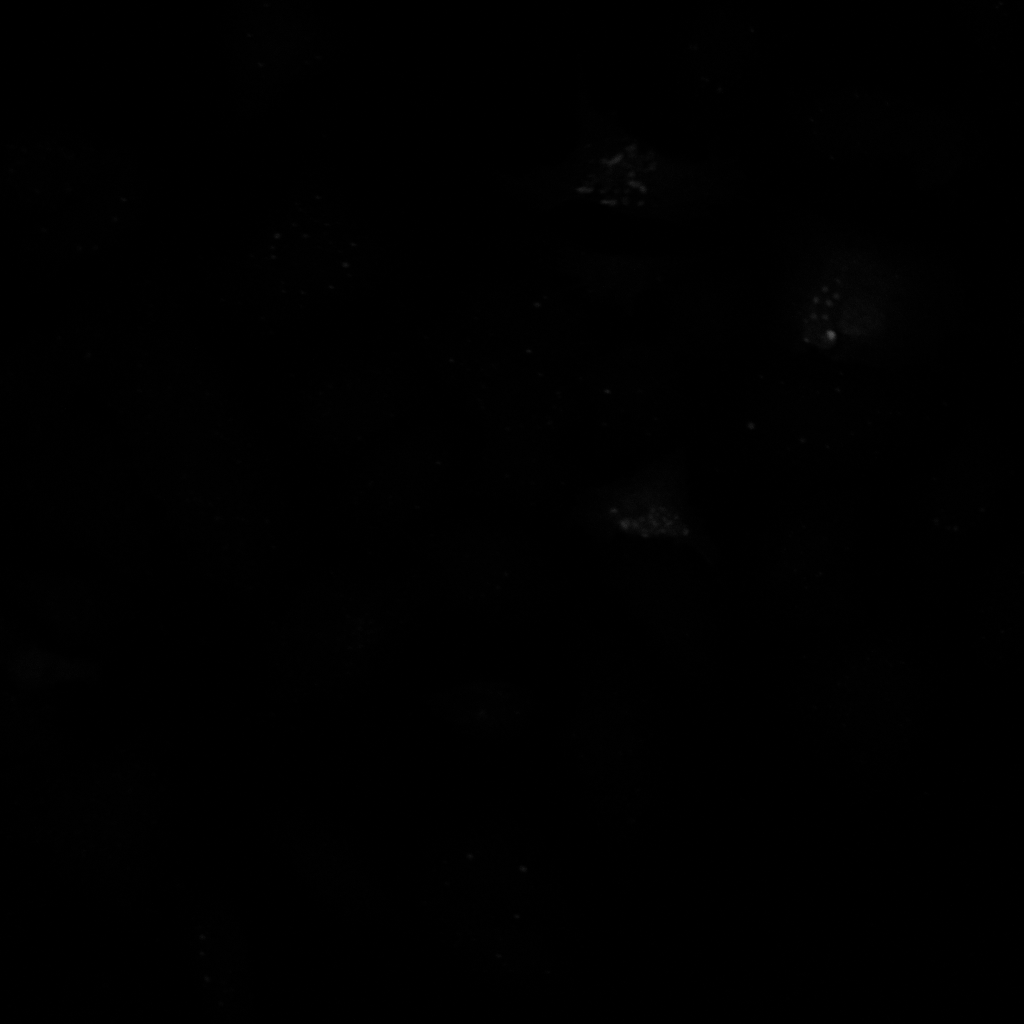

Supplement: Supplementary file 2 — Supplementary Information 1. [file 41598_2024_63884_MOESM2_ESM.zip › Additional_file_1/HAP40-TS/series 002_c3.tif]

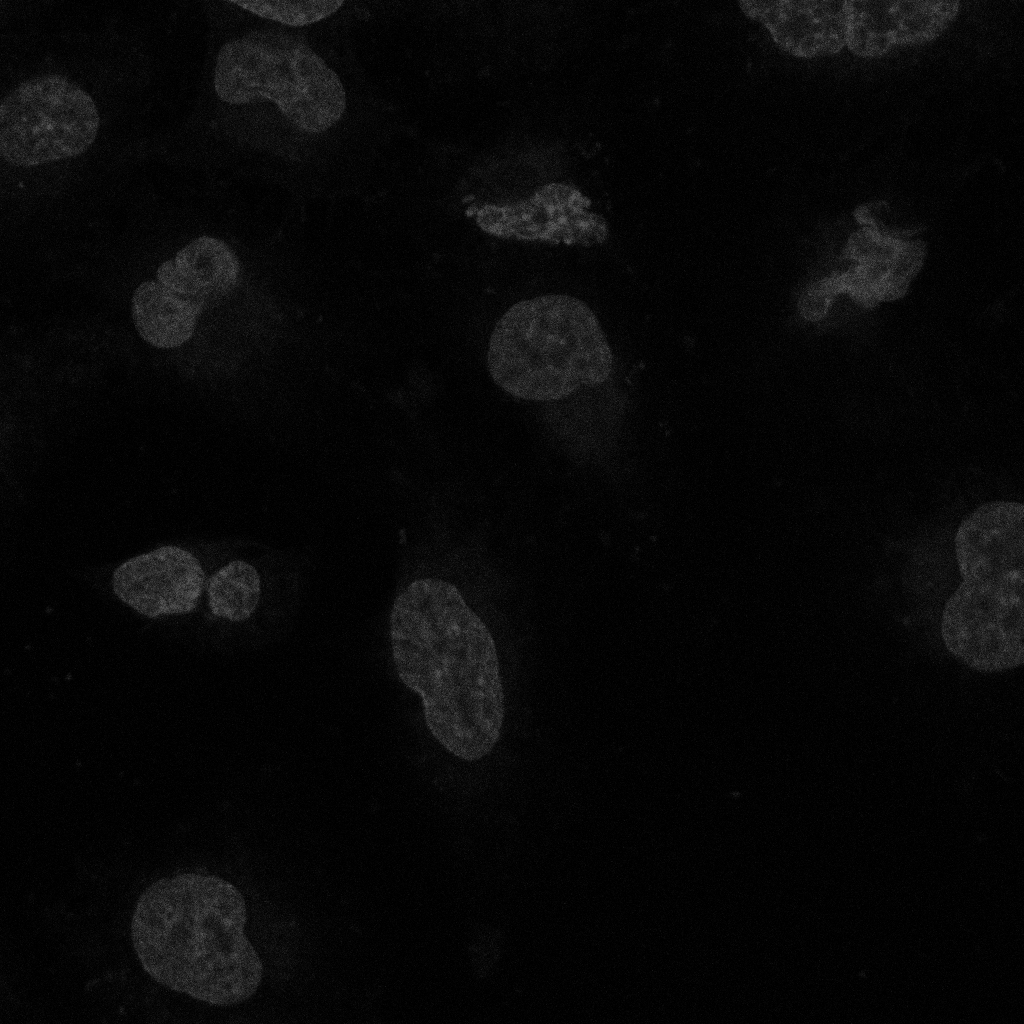

Supplement: Supplementary file 2 — Supplementary Information 1. [file 41598_2024_63884_MOESM2_ESM.zip › Additional_file_1/HAP40-TS/series 009_c1.tif]

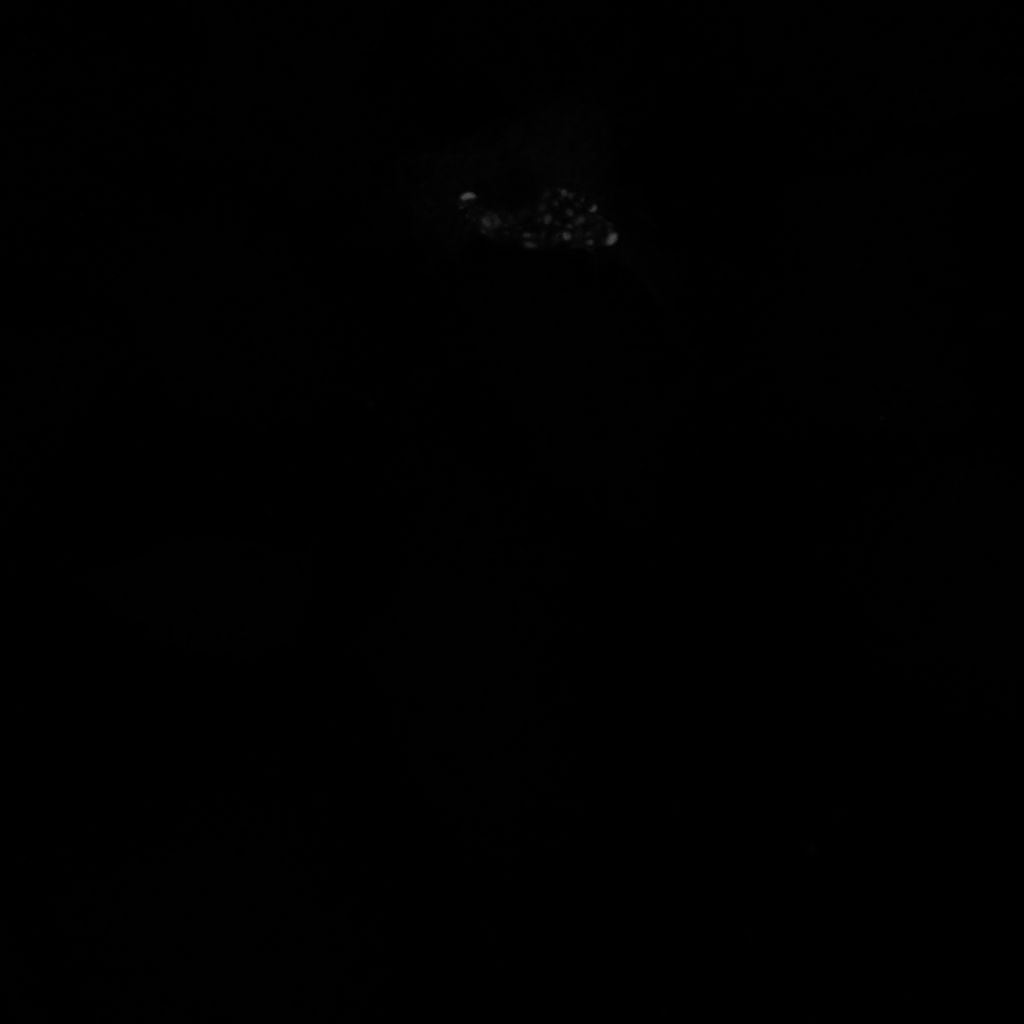

Supplement: Supplementary file 2 — Supplementary Information 1. [file 41598_2024_63884_MOESM2_ESM.zip › Additional_file_1/HAP40-TS/series 009_c2.tif]

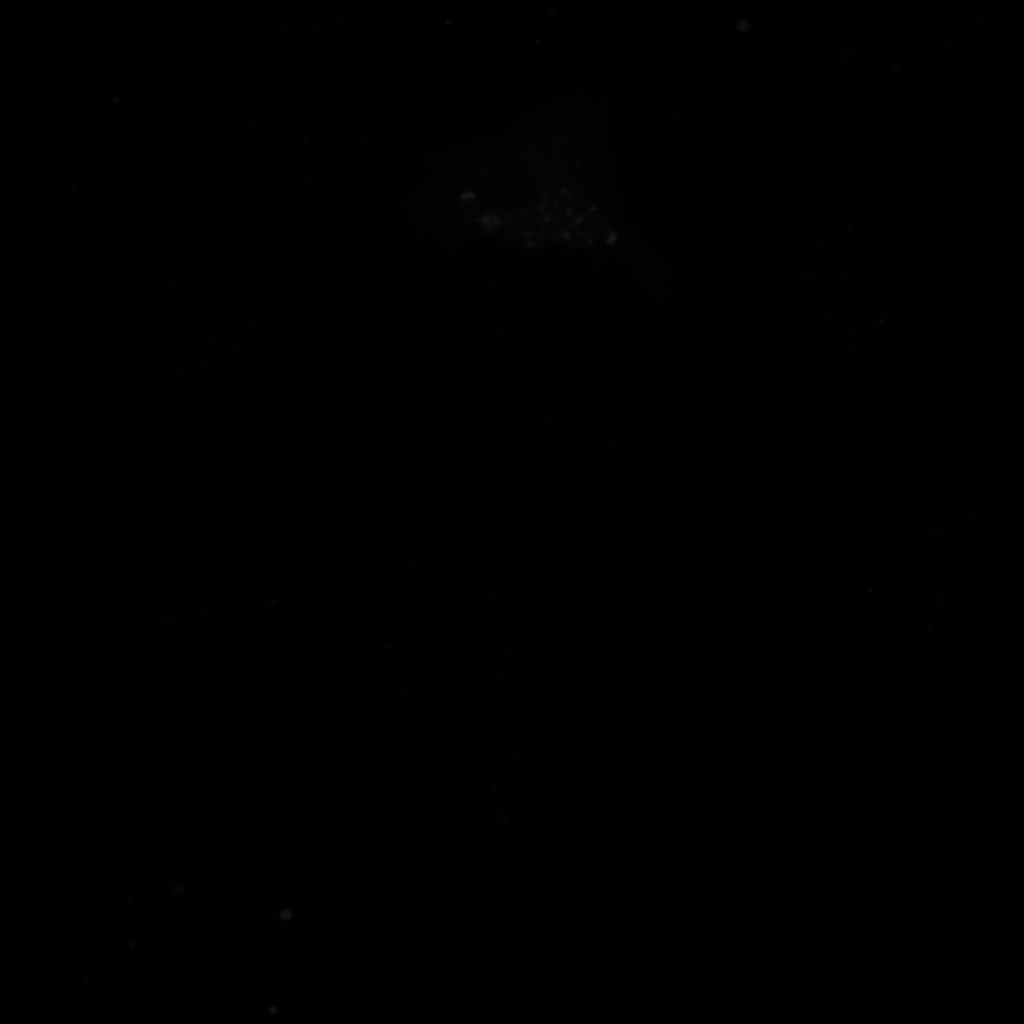

Supplement: Supplementary file 2 — Supplementary Information 1. [file 41598_2024_63884_MOESM2_ESM.zip › Additional_file_1/HAP40-TS/series 009_c3.tif]

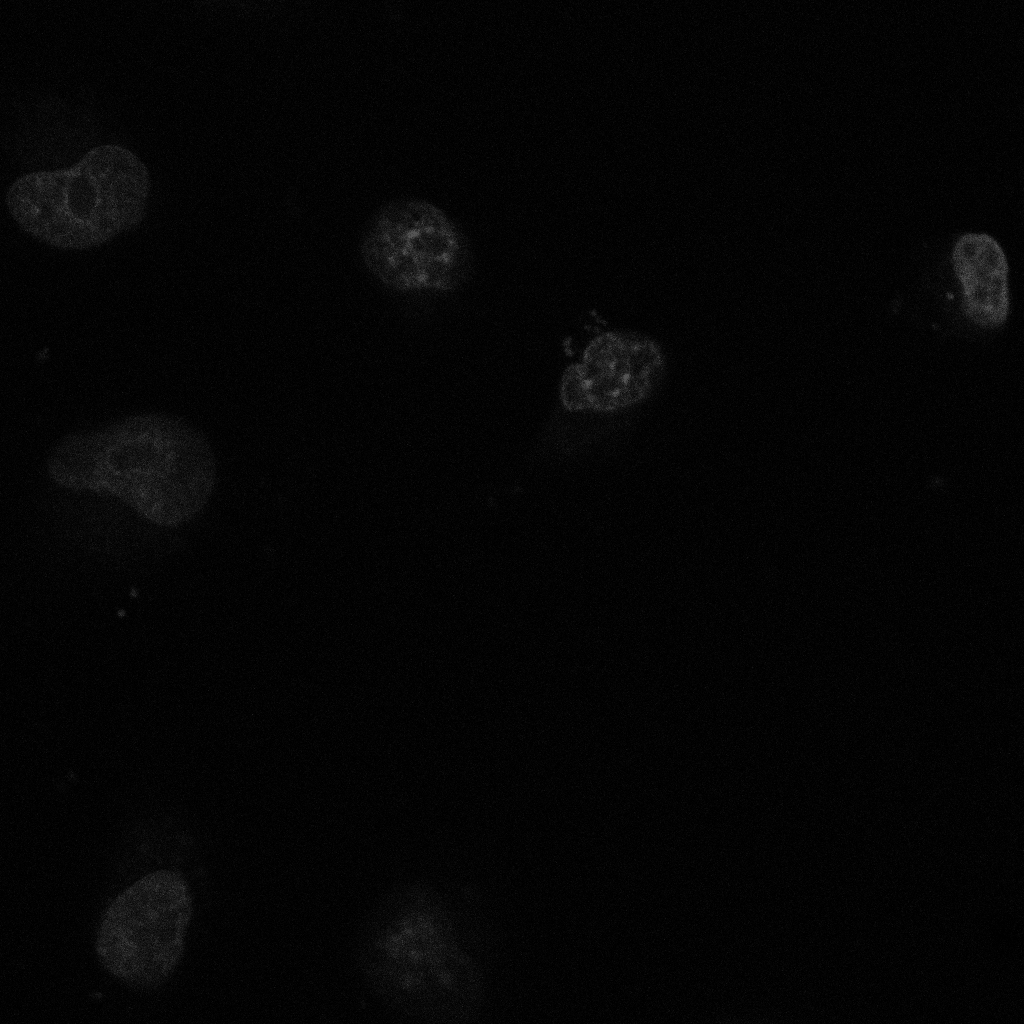

Supplement: Supplementary file 2 — Supplementary Information 1. [file 41598_2024_63884_MOESM2_ESM.zip › Additional_file_1/HAP40-TS/series 011_c1.tif]

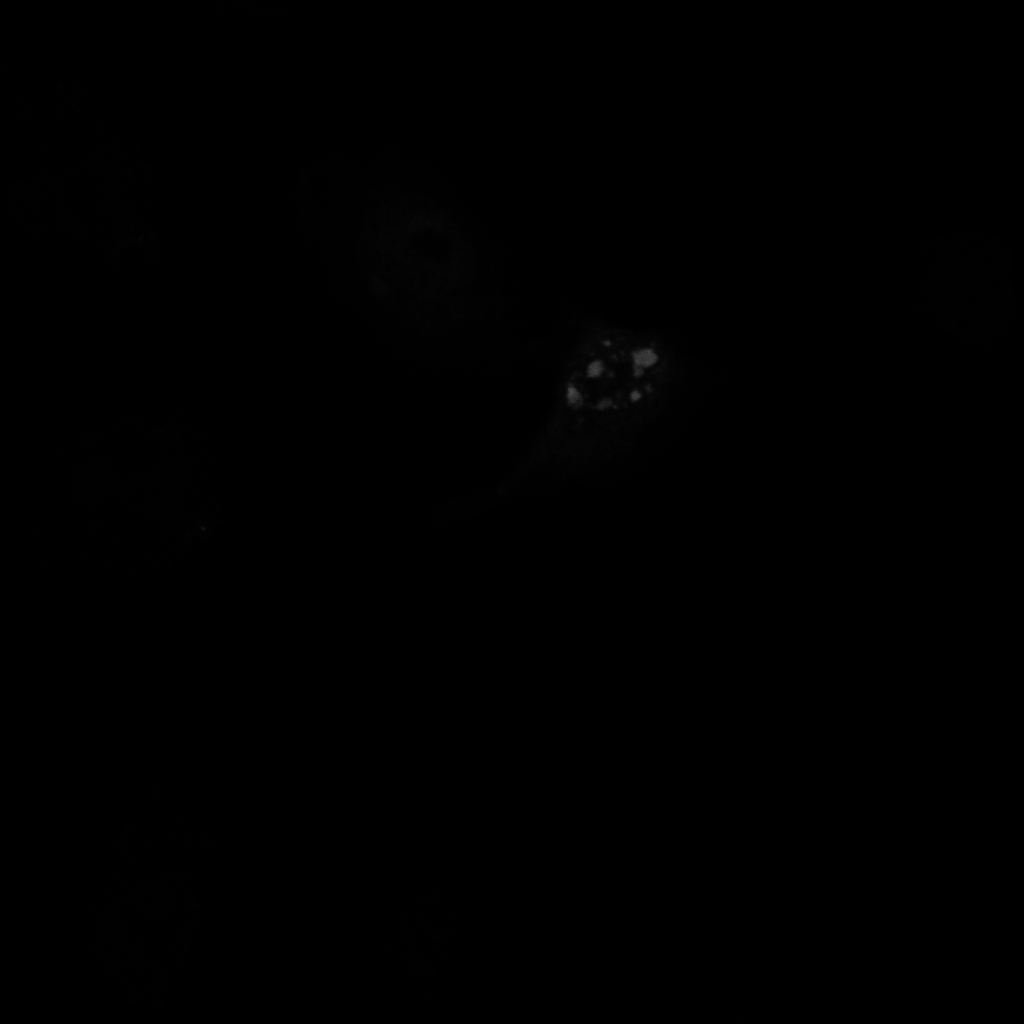

Supplement: Supplementary file 2 — Supplementary Information 1. [file 41598_2024_63884_MOESM2_ESM.zip › Additional_file_1/HAP40-TS/series 011_c2.tif]

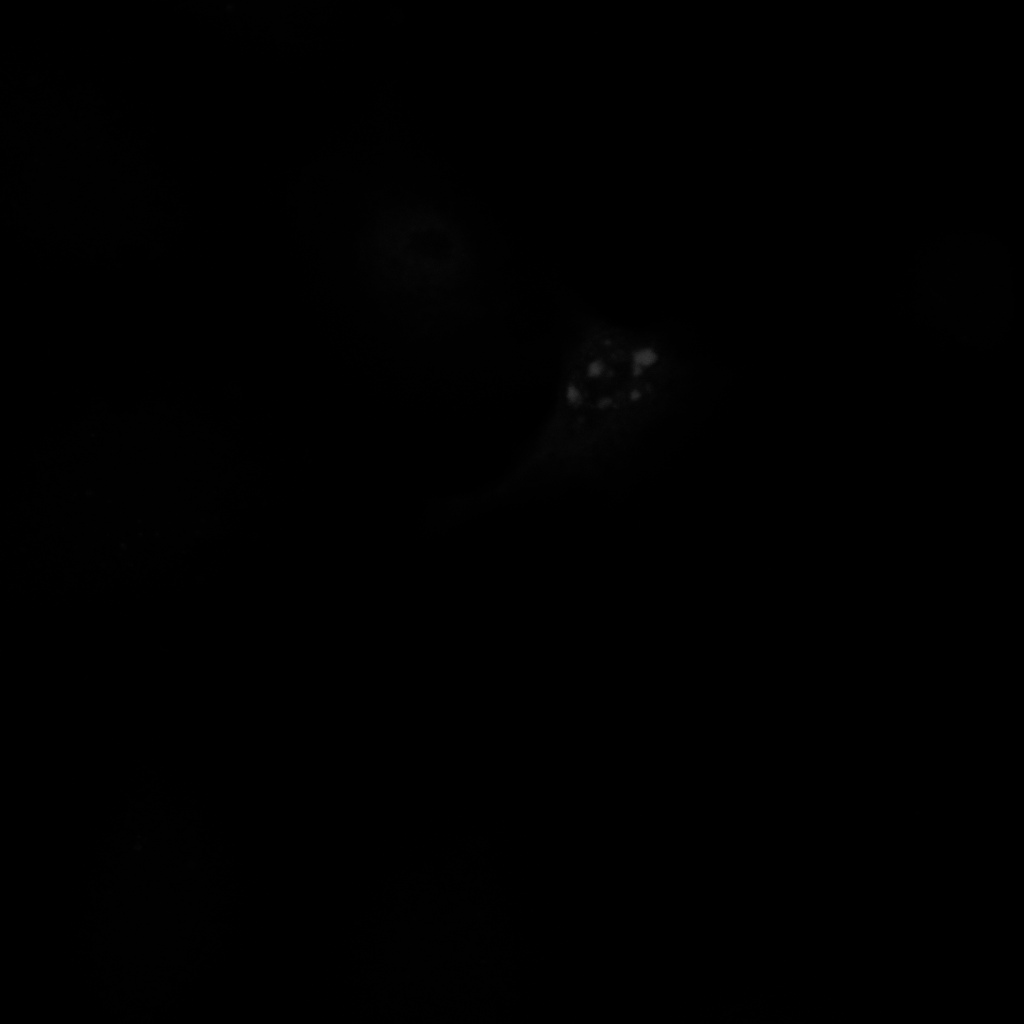

Supplement: Supplementary file 2 — Supplementary Information 1. [file 41598_2024_63884_MOESM2_ESM.zip › Additional_file_1/HAP40-TS/series 011_c3.tif]

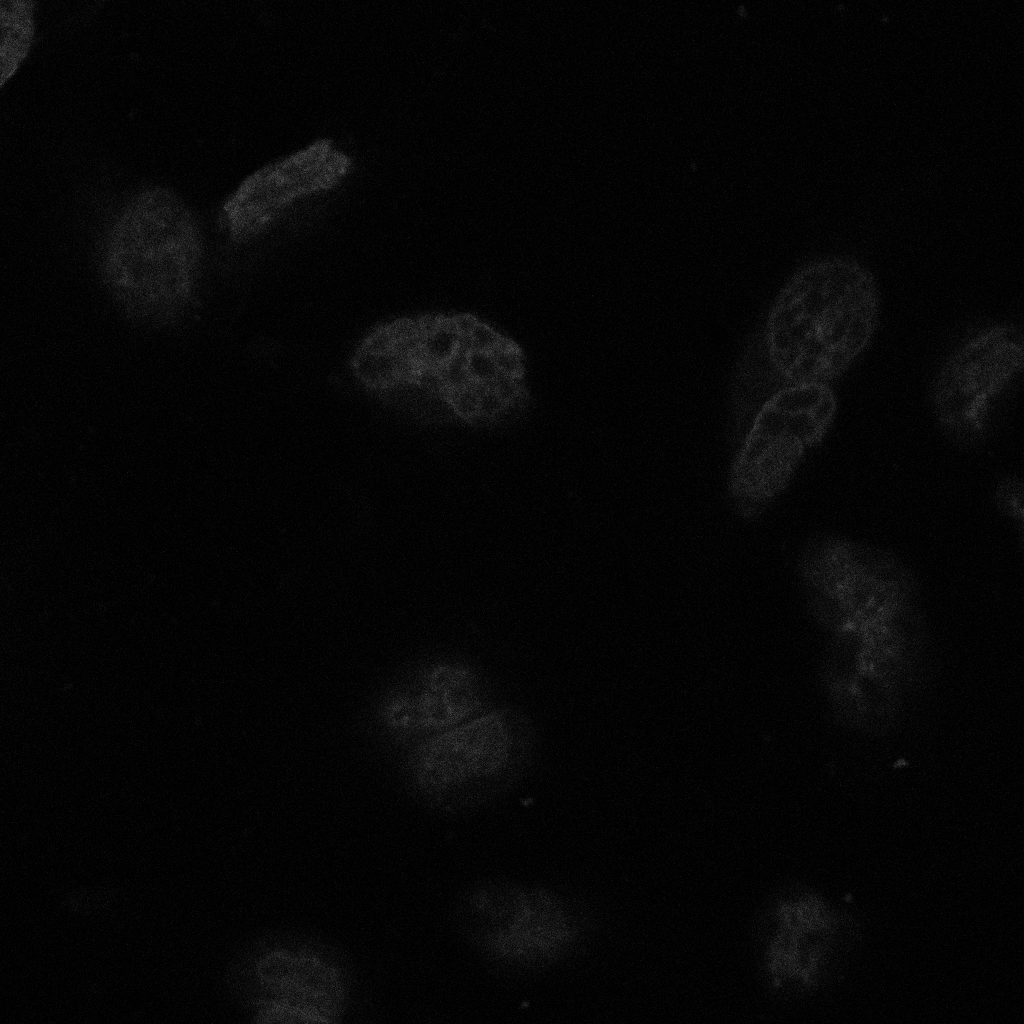

Supplement: Supplementary file 2 — Supplementary Information 1. [file 41598_2024_63884_MOESM2_ESM.zip › Additional_file_1/HAP40-TS/series 014_c1.tif]

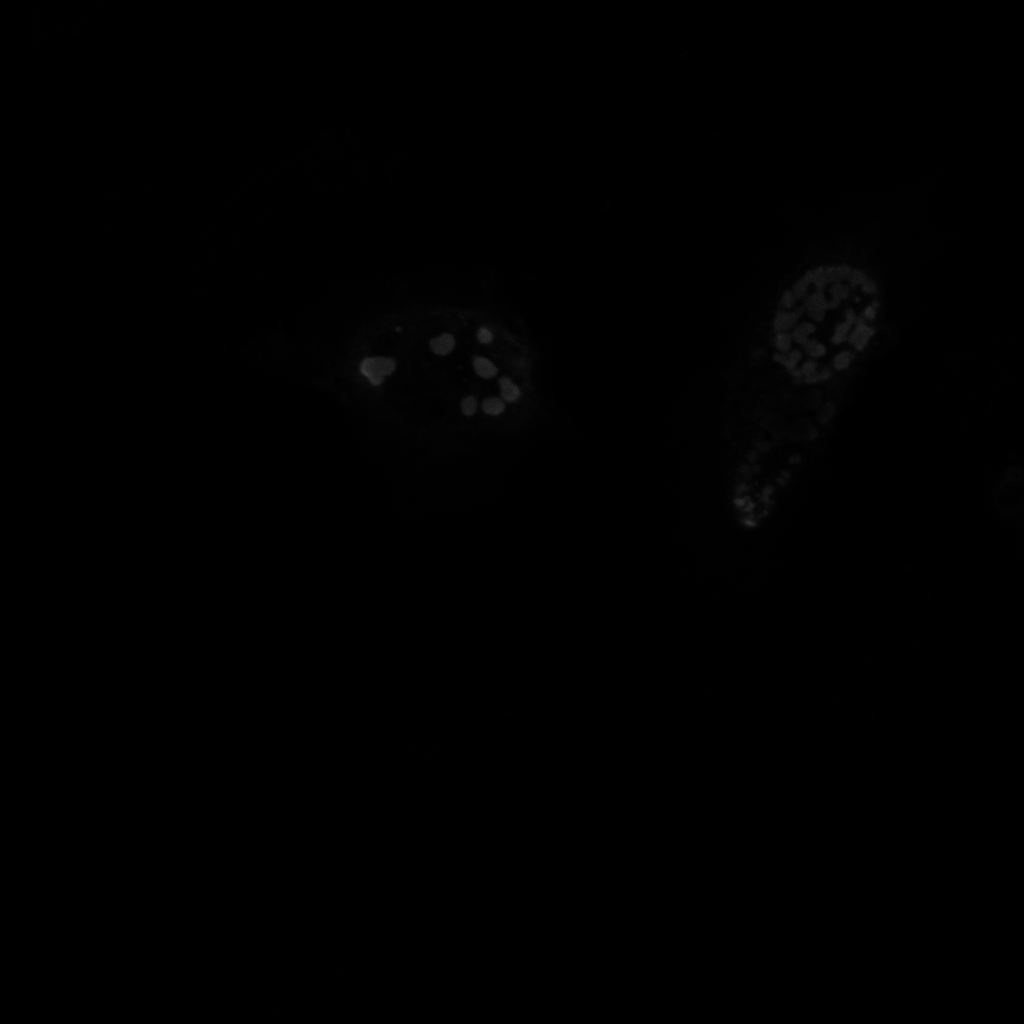

Supplement: Supplementary file 2 — Supplementary Information 1. [file 41598_2024_63884_MOESM2_ESM.zip › Additional_file_1/HAP40-TS/series 014_c2.tif]

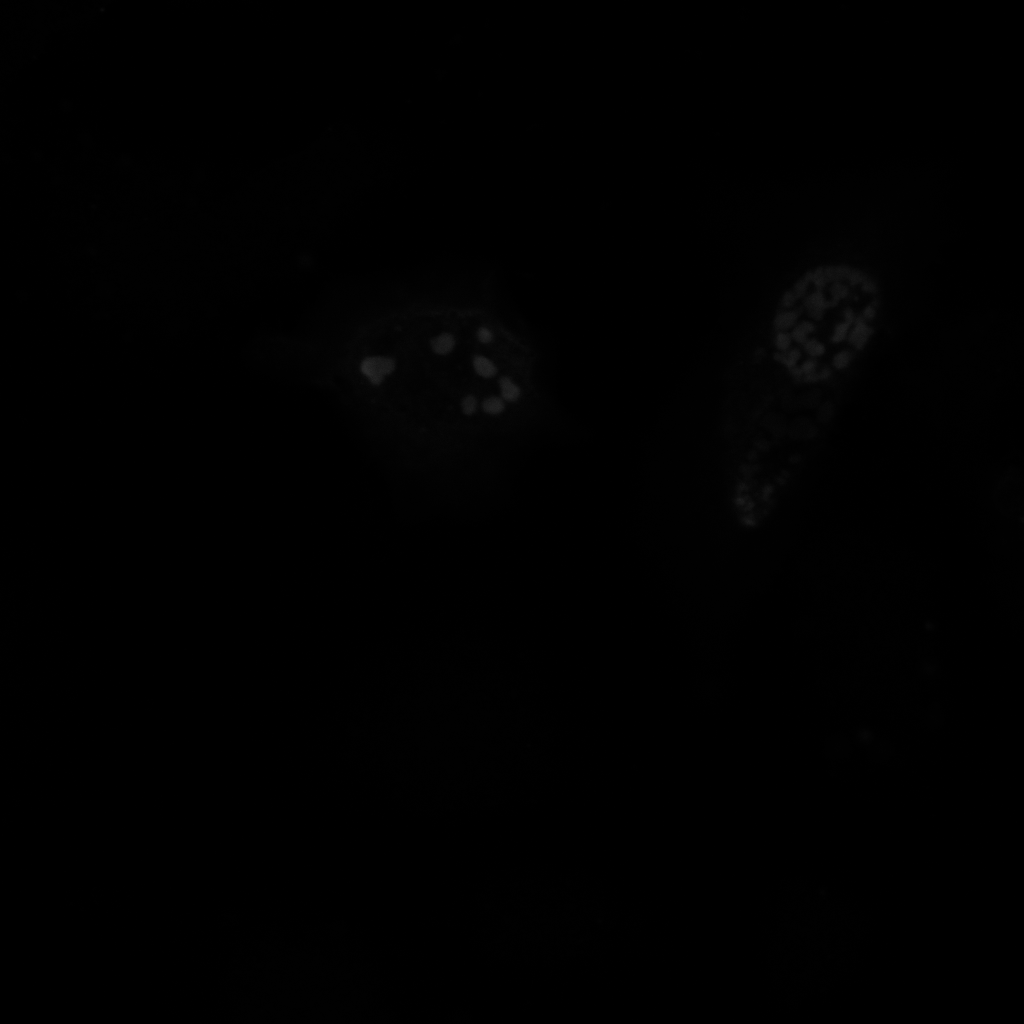

Supplement: Supplementary file 2 — Supplementary Information 1. [file 41598_2024_63884_MOESM2_ESM.zip › Additional_file_1/HAP40-TS/series 014_c3.tif]

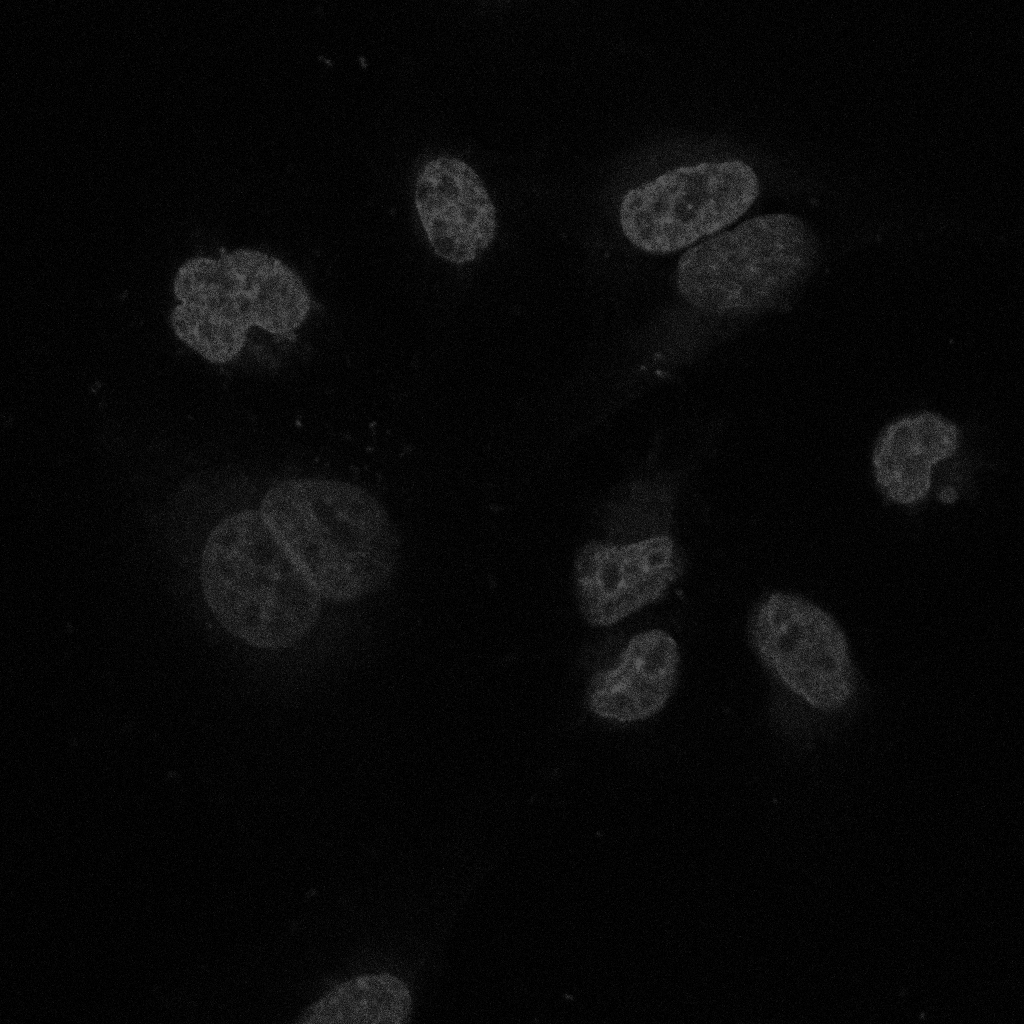

Supplement: Supplementary file 2 — Supplementary Information 1. [file 41598_2024_63884_MOESM2_ESM.zip › Additional_file_1/HAP40-TS/series 016_c1.tif]

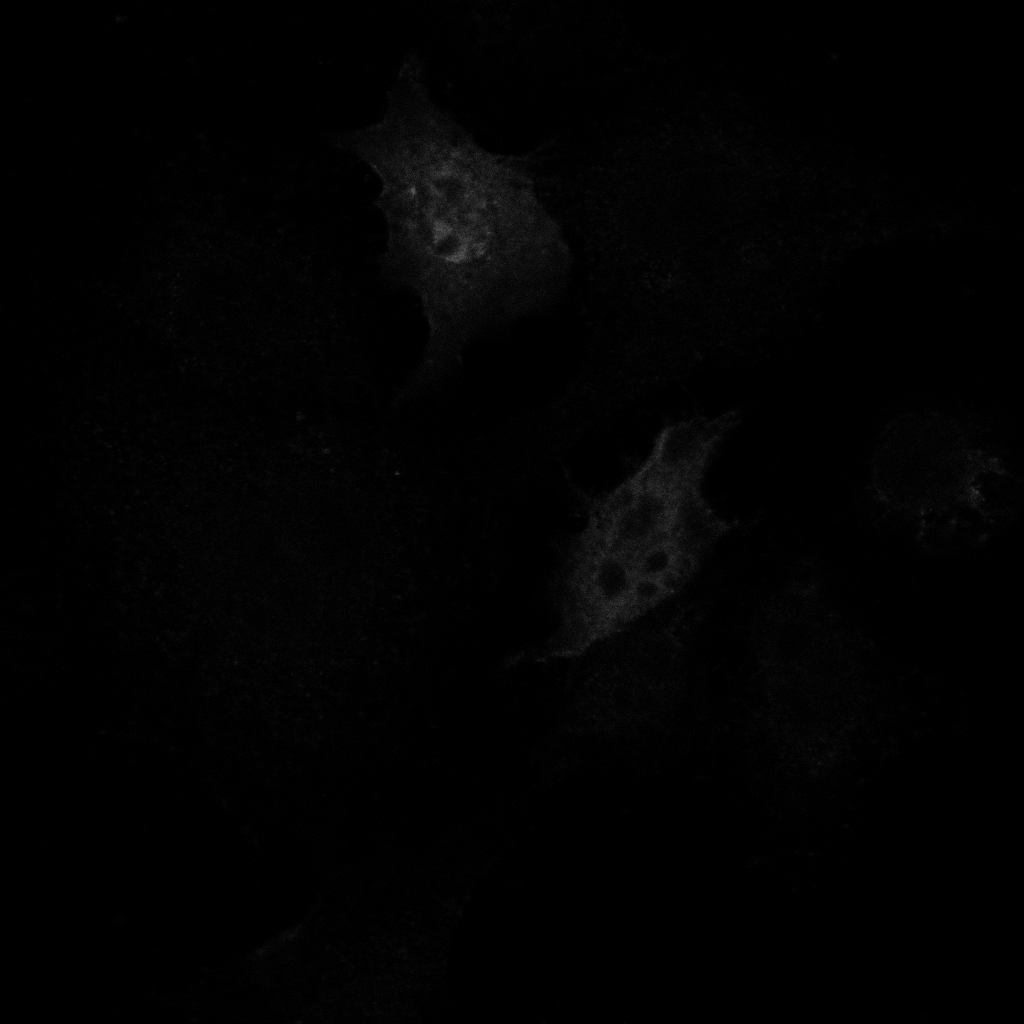

Supplement: Supplementary file 2 — Supplementary Information 1. [file 41598_2024_63884_MOESM2_ESM.zip › Additional_file_1/HAP40-TS/series 016_c2.tif]

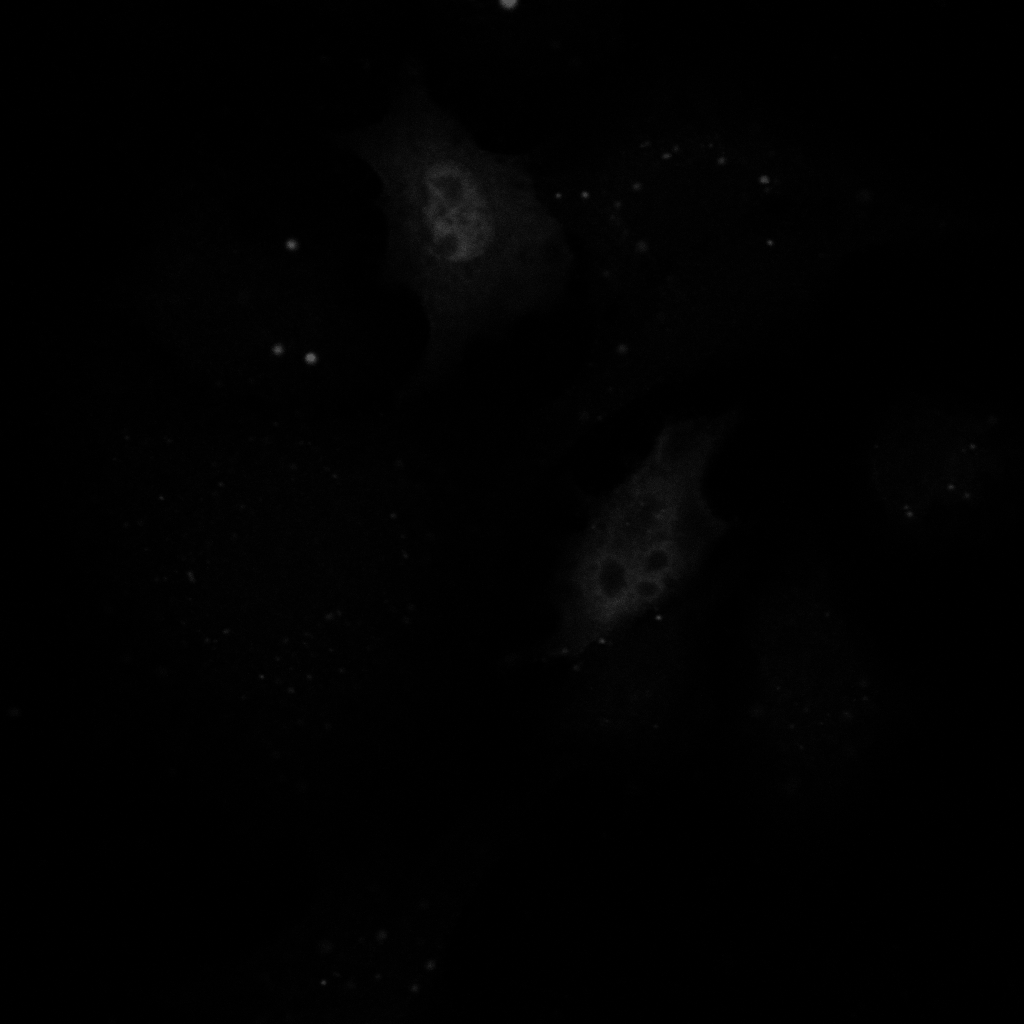

Supplement: Supplementary file 2 — Supplementary Information 1. [file 41598_2024_63884_MOESM2_ESM.zip › Additional_file_1/HAP40-TS/series 016_c3.tif]

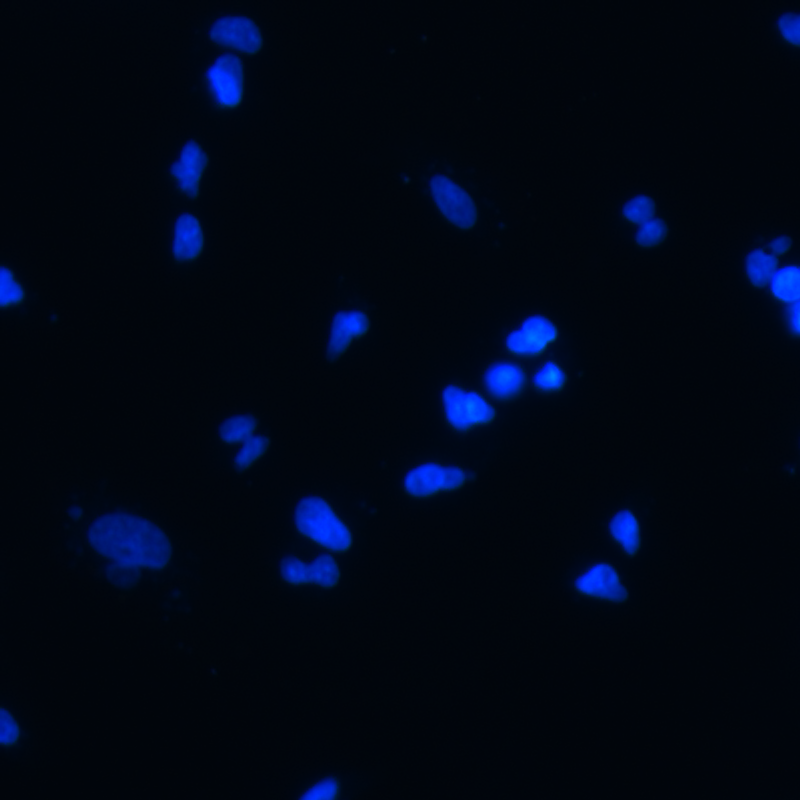

Supplement: Supplementary file 3 — Supplementary Information 2. [file 41598_2024_63884_MOESM3_ESM.zip › 0001_c1.tif]

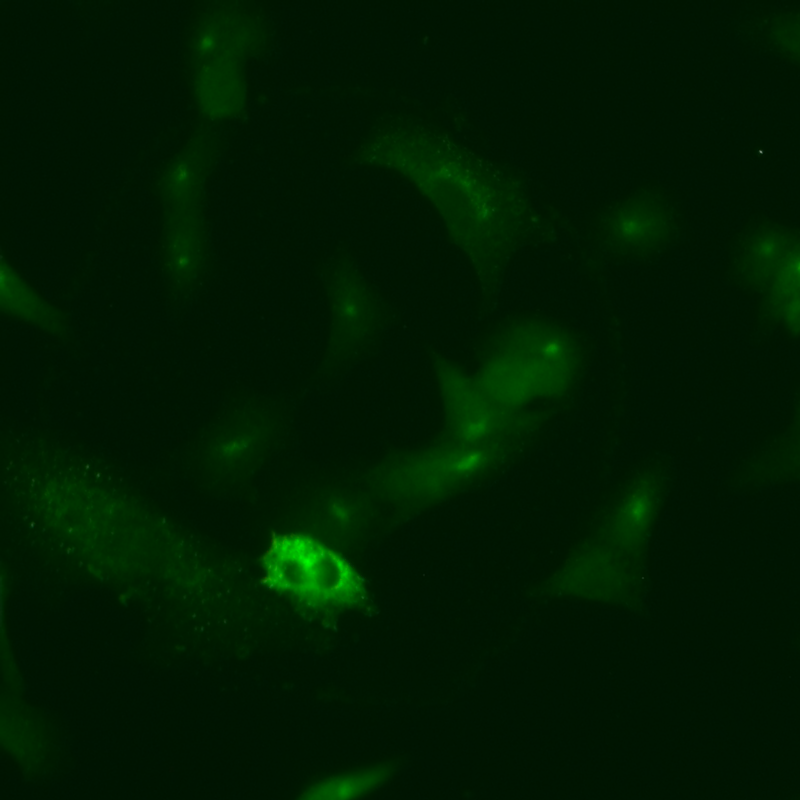

Supplement: Supplementary file 3 — Supplementary Information 2. [file 41598_2024_63884_MOESM3_ESM.zip › 0001_c2.tif]

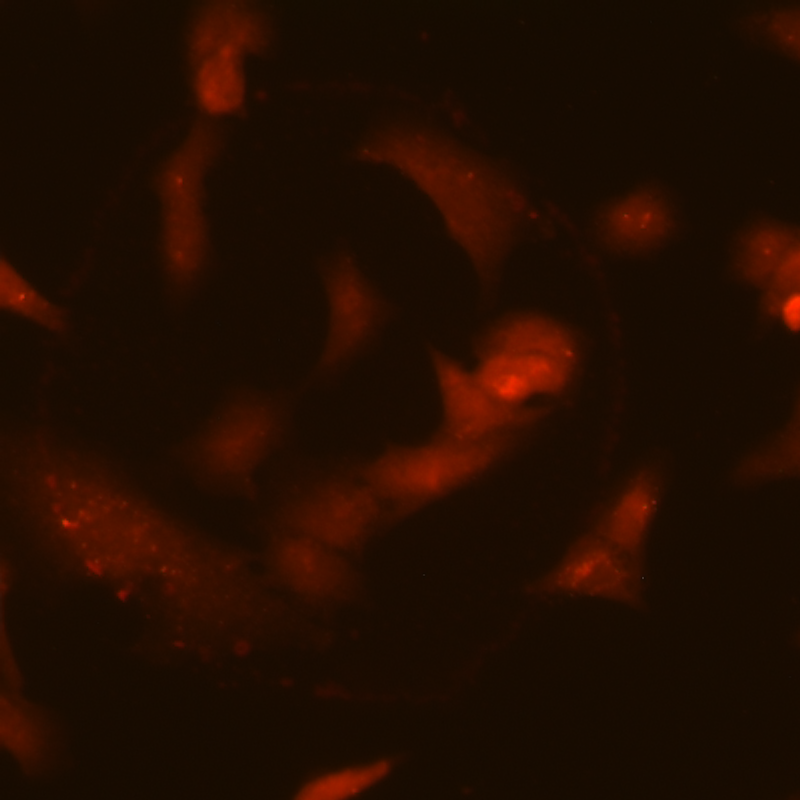

Supplement: Supplementary file 3 — Supplementary Information 2. [file 41598_2024_63884_MOESM3_ESM.zip › 0001_c3.tif]

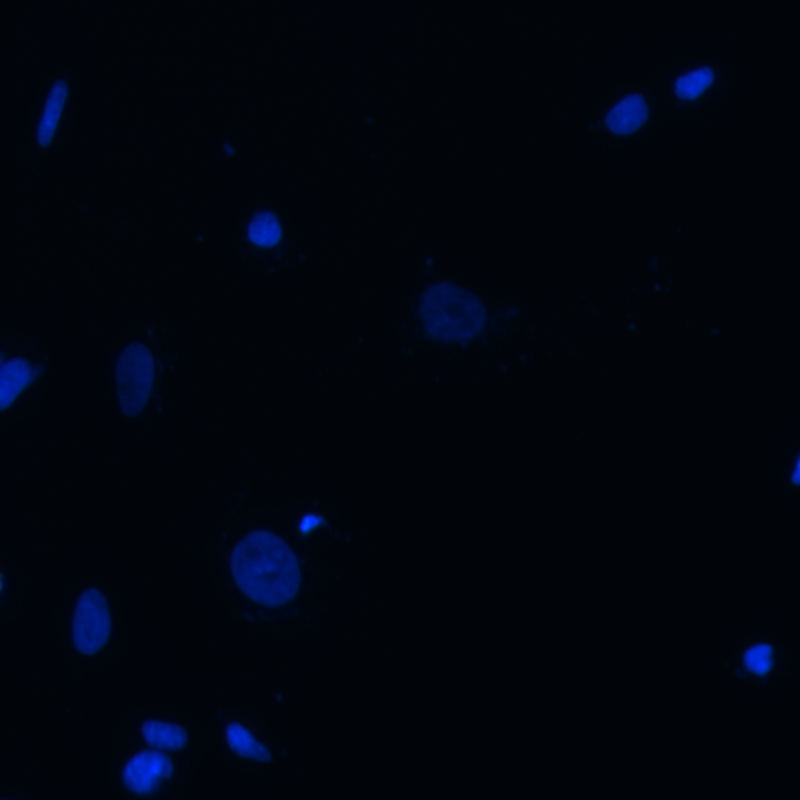

Supplement: Supplementary file 3 — Supplementary Information 2. [file 41598_2024_63884_MOESM3_ESM.zip › 0002_c1.tif]

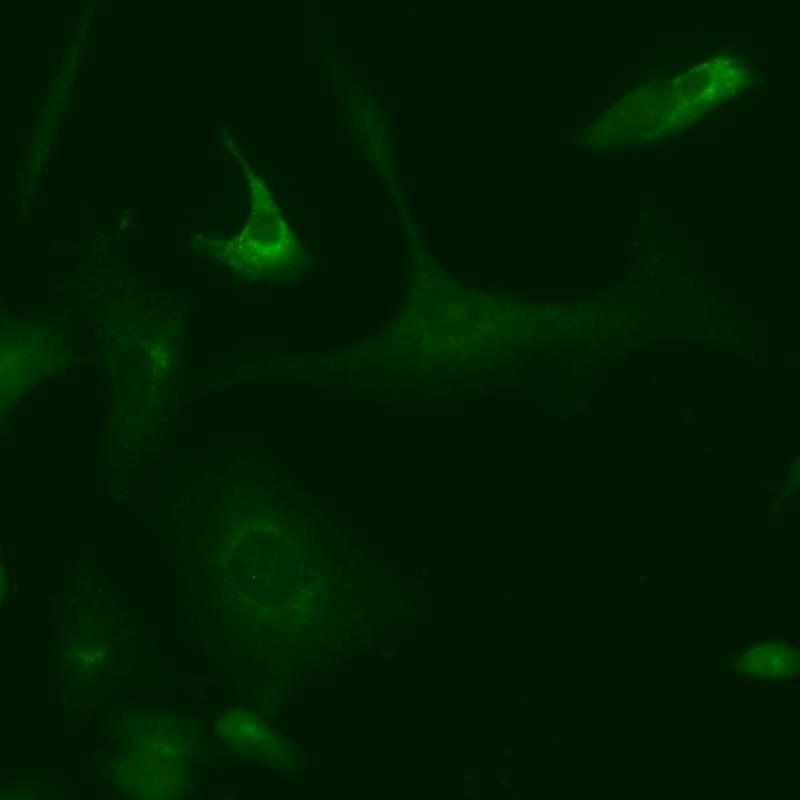

Supplement: Supplementary file 3 — Supplementary Information 2. [file 41598_2024_63884_MOESM3_ESM.zip › 0002_c2.tif]

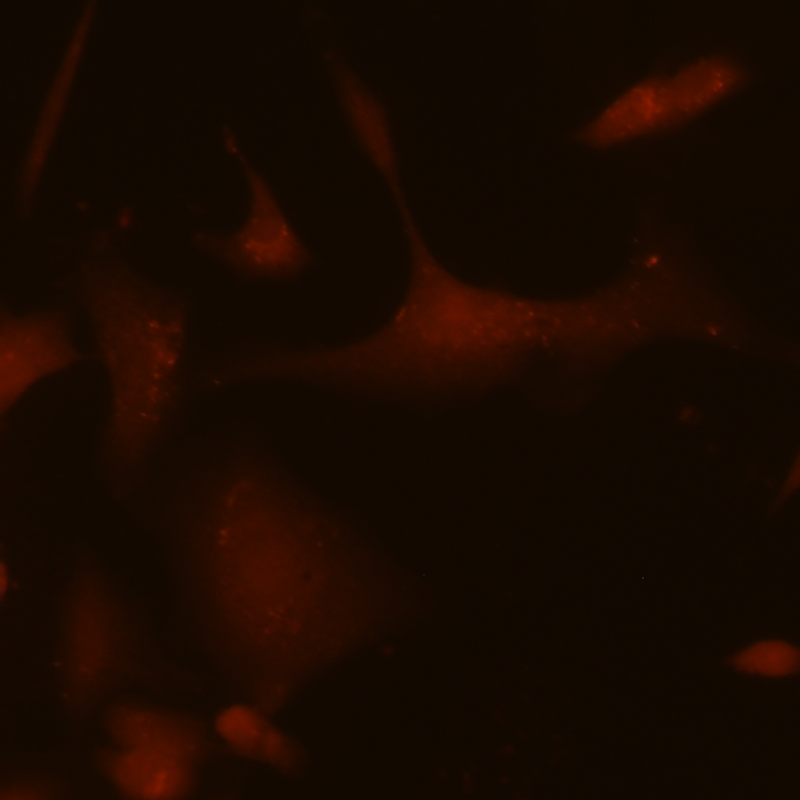

Supplement: Supplementary file 3 — Supplementary Information 2. [file 41598_2024_63884_MOESM3_ESM.zip › 0002_c3.tif]

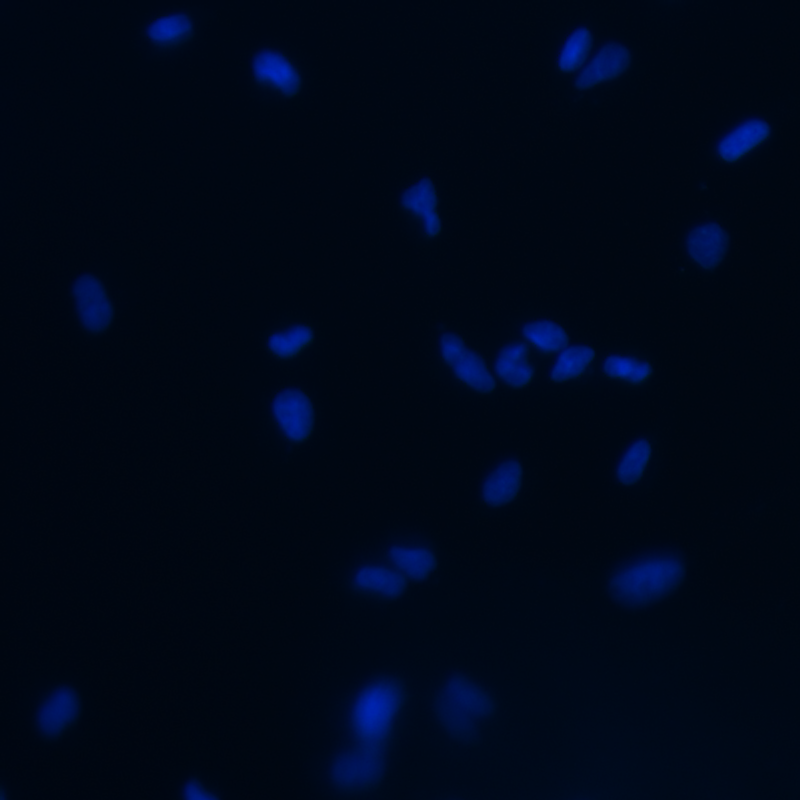

Supplement: Supplementary file 3 — Supplementary Information 2. [file 41598_2024_63884_MOESM3_ESM.zip › 0003_c1.tif]

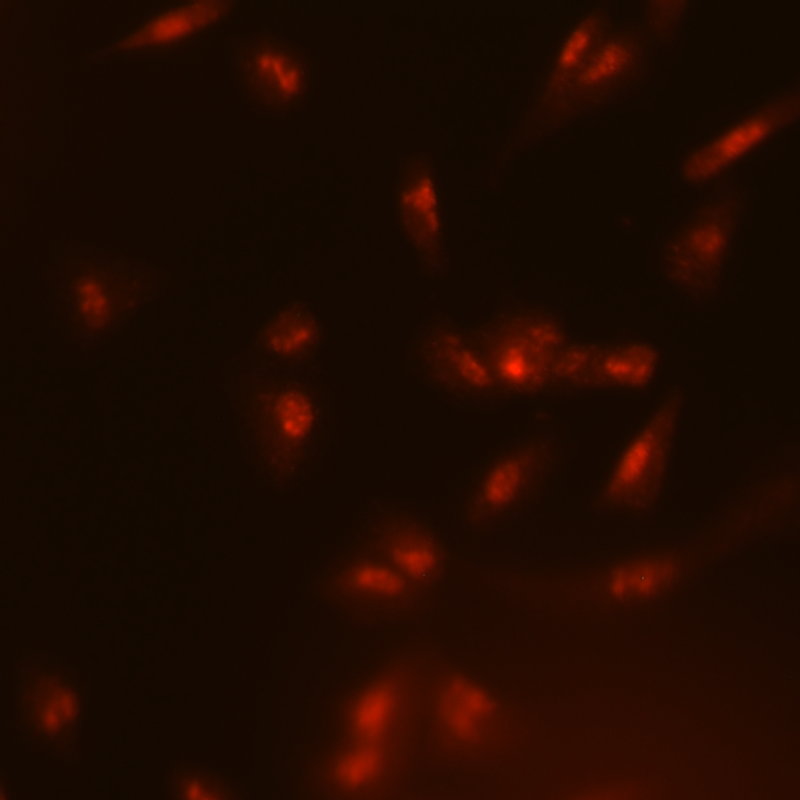

Supplement: Supplementary file 3 — Supplementary Information 2. [file 41598_2024_63884_MOESM3_ESM.zip › 0003_c2.tif]

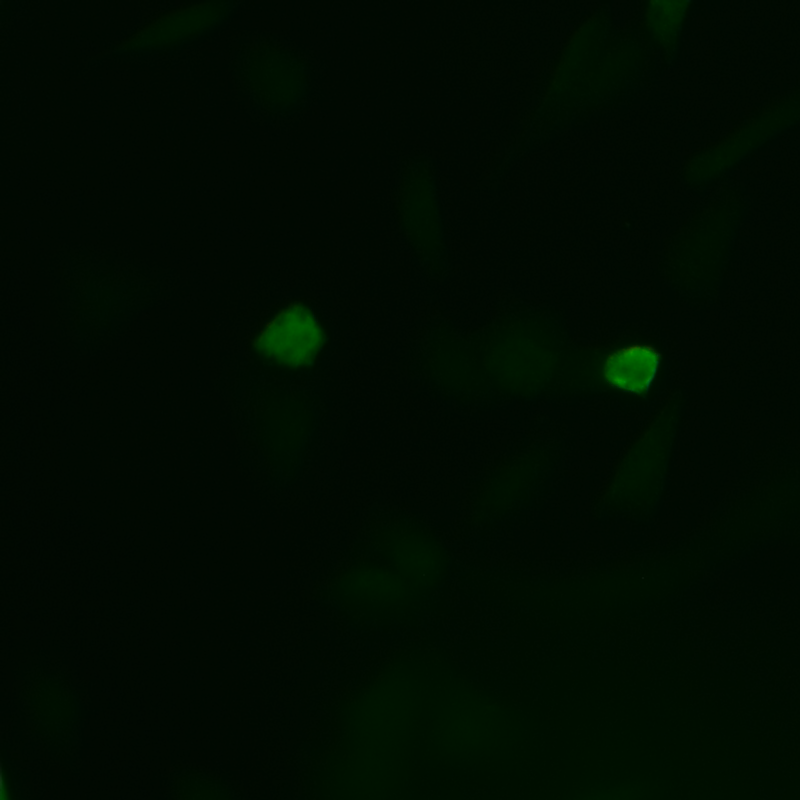

Supplement: Supplementary file 3 — Supplementary Information 2. [file 41598_2024_63884_MOESM3_ESM.zip › 0003_c3.tif]

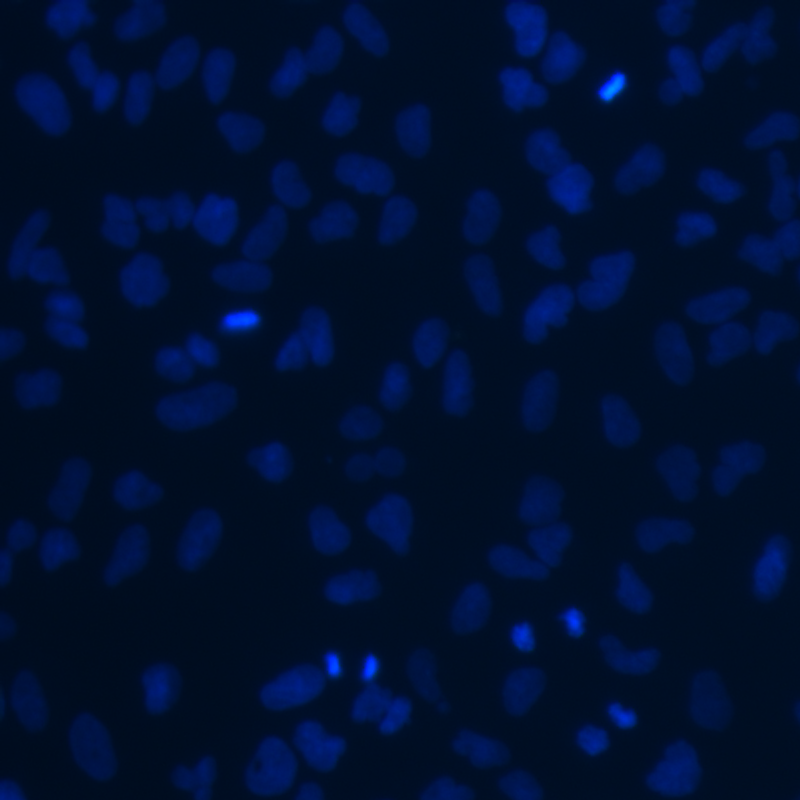

Supplement: Supplementary file 3 — Supplementary Information 2. [file 41598_2024_63884_MOESM3_ESM.zip › 0004_c1.tif]

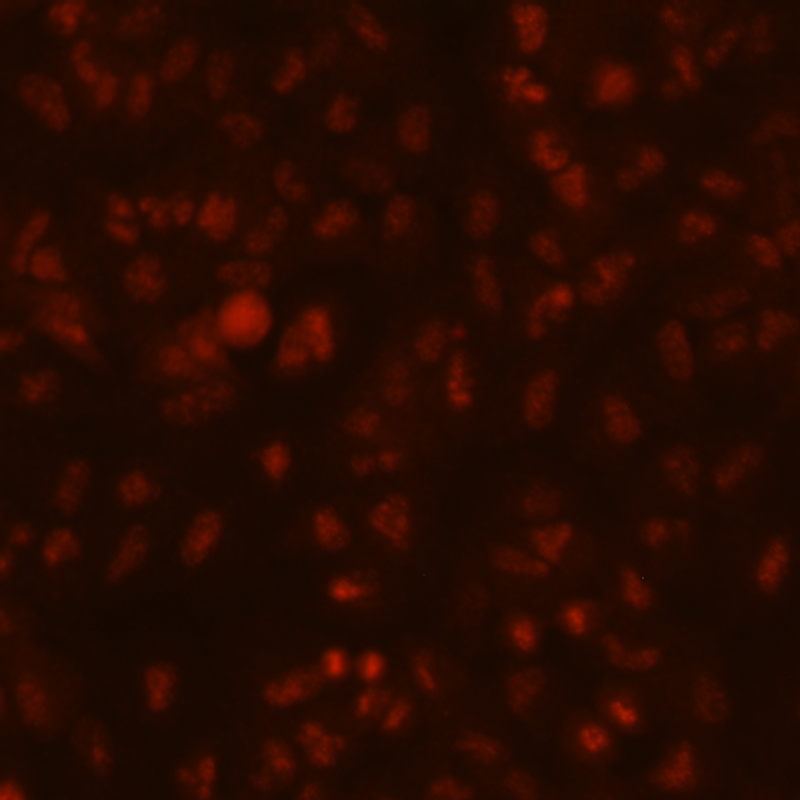

Supplement: Supplementary file 3 — Supplementary Information 2. [file 41598_2024_63884_MOESM3_ESM.zip › 0004_c2.tif]

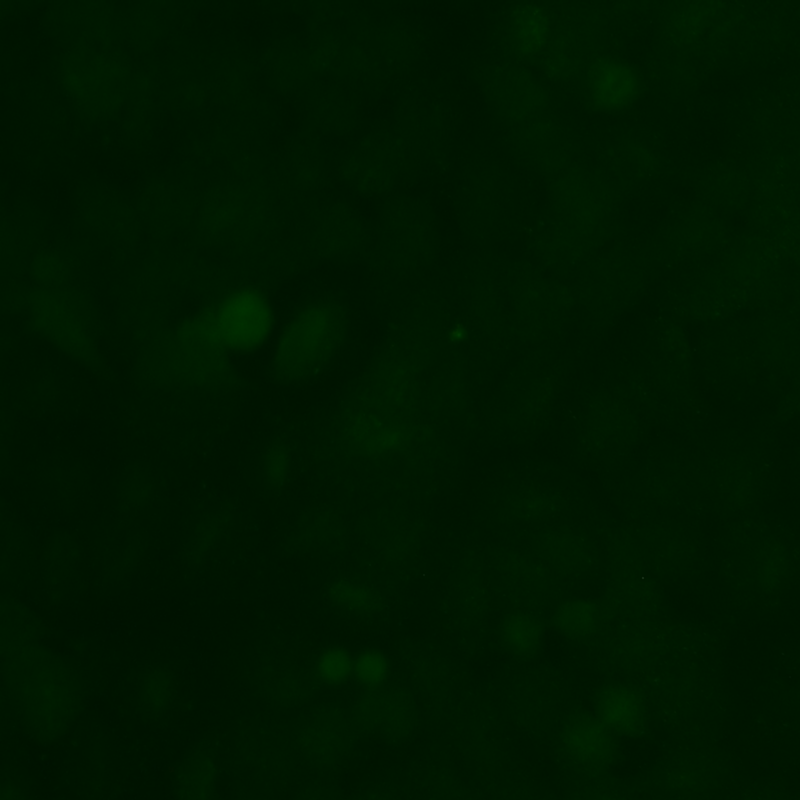

Supplement: Supplementary file 3 — Supplementary Information 2. [file 41598_2024_63884_MOESM3_ESM.zip › 0004_c3.tif]
