# Supplementary material for: ProteinCoLoc streamlines Bayesian analysis of colocalization in microscopic images
Source: Sci Rep. 2024 Jun 10;14:13277. doi: 10.1038/s41598-024-63884-1 (PMC11164984; doi:10.1038/s41598-024-63884-1)
Supplement: Supplementary file 5 — Supplementary Information 4. [file 41598_2024_63884_MOESM5_ESM.docx]

**Supplementary file S4: Step-by-step guide**

In the following, we provide a detailed, step-by-step walkthrough on how to effectively use the graphical user interface (GUI) of ProteinCoLoc. For the purposes of this example, we will be utilizing the confocal images provided in Supplementary File S1. To assist with navigating the interface, informative tooltips are readily available — simply hover over any setting or option, and a tooltip will appear, offering additional details and insights about its function.

1. **Install ProteinCoLoc:**
   1. Download the compiled version of ProteinCoLoc.Launch the application by executing the “ProteinCoLoc.exe” file located in the base directory of ProteinCoLoc.
   2. The initial startup of ProteinCoLoc may take up to five minutes and requires an internet connection to download various components (Julia artifacts) necessary for operation. **Note:** a Glib-GIO-WARNING message may appear in the terminal during the start of ProteinCoLoc. This warning message can be safely ignored as it does not impact ProteinCoLoc.
2. **Prepare the images for analysis:**
   1. **Images from Supplementary file S1:** Download and unzip Supplementary file S1. Download and unzip the Supplementary File S1. Transfer the unzipped folders to a designated directory for analysis.
   2. **Own images:**
      1. Generate two separate folders, one for each biological group you are analyzing.
      2. Ensure that image files within these folders adhere to the naming convention outlined in the publication. For instance, images of channels 1, 2, and 3 from sample S1 should be named S1_c1.tiff, S1_c2.tiff, and S1_c3.tiff, respectively.
3. **Generate an empty directory to store the results of the analysis.** Note: ProteinCoLoc performs a preliminary check to determine if the specified directory contains data from a previous analysis. This safety measure is in place to prevent the overwriting of existing results. If ProteinCoLoc detects pre-existing data, it will halt execution.
4. **Copy the path of these folders into the GUI**


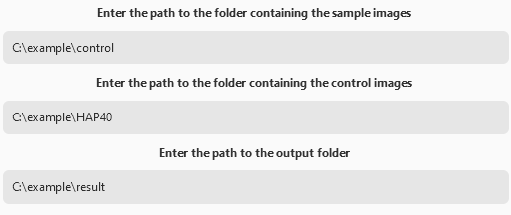


1. **Adjust the number of recorded color channels to 3**


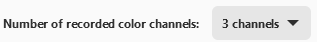


1. We will only compute the colocalisation between color channels 2 and 3. Therefore, we deactivate the toggle “All combinations” and replace the text in the text field below “Only two channels” to 2,3.


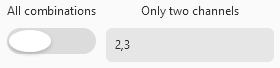


1. Furthermore, we will employ a stricter threshold for protein colocalisation then the default. Therefore, we change the $\Delta\rho$ threshold to 0.3.


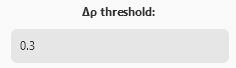


1. **Initiate the analysis by clicking on the “Start Analysis” button.** Please be aware that during the analysis process, the application window may become unresponsive and appear to freeze. This is normal and does not indicate a problem; the window will become responsive again once the analysis is complete.
   1. Monitoring Progress: The terminal will display the progress of the analysis along with any error messages that may arise. It's important to keep an eye on the terminal for updates or potential issues during the analysis.
   2. Reviewing Results and Logs: Upon completion, the results will be stored in the previously specified results directory. Alongside the results, a comprehensive log file is also generated, documenting the configuration of the analysis and any noteworthy events that occurred. This log file is invaluable for verifying the analysis parameters and troubleshooting any issues.
