# Supplementary material for: ProteinCoLoc streamlines Bayesian analysis of colocalization in microscopic images
Source: Sci Rep. 2024 Jun 10;14:13277. doi: 10.1038/s41598-024-63884-1 (PMC11164984; doi:10.1038/s41598-024-63884-1)
Supplement: Supplementary file 6 — Supplementary Information 5. [file 41598_2024_63884_MOESM6_ESM.docx]

**Supplementary file S5: Runtime and space complexity.**


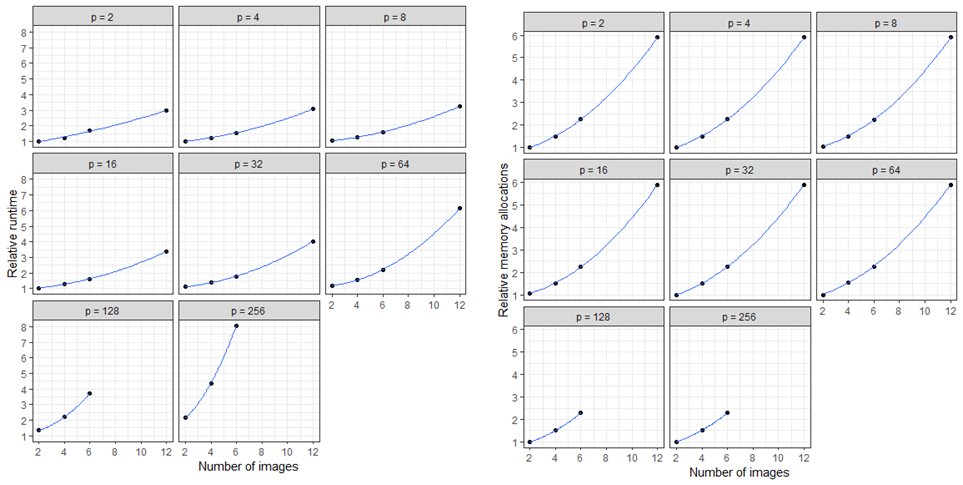


**Figure 1: Runtime and space complexity of ProteinCoLoc.** Runtime and memory allocations are displayed relative to the measured minimum with 2 images and 2 patches / per image. Runtime and memory allocations were determined with Julia’s built-in macro @time.
